# Palmitoylated APP Forms Dimers, Cleaved by BACE1

**DOI:** 10.1371/journal.pone.0166400

**Published:** 2016-11-22

**Authors:** Raja Bhattacharyya, Rebecca H. Fenn, Cory Barren, Rudolph E. Tanzi, Dora M. Kovacs

**Affiliations:** Genetics and Aging Research Unit, MassGeneral Institute for Neurodegenerative Diseases (MIND), Massachusetts General Hospital, Harvard Medical School, Charlestown, MA 02129, United States of America; National Center for Geriatrics and Gerontology, JAPAN

## Abstract

A major rate-limiting step for Aβ generation and deposition in Alzheimer’s disease brains is BACE1-mediated cleavage (β-cleavage) of the amyloid precursor protein (APP). We previously reported that APP undergoes palmitoylation at two cysteine residues (Cys^186^ and Cys^187^) in the E1-ectodomain. 8–10% of total APP is palmitoylated *in vitro* and *in vivo*. Palmitoylated APP (*pal*APP) shows greater preference for β-cleavage than total APP in detergent resistant lipid rafts. Protein palmitoylation is known to promote protein dimerization. Since dimerization of APP at its E1-ectodomain results in elevated BACE1-mediated cleavage of APP, we have now investigated whether palmitoylation of APP affects its dimerization and whether this leads to elevated β-cleavage of the protein. Here we report that over 90% of *pal*APP is dimerized while only ~20% of total APP forms dimers. *Pal*APP-dimers are predominantly *cis*-oriented while total APP dimerizes in both *cis*- and *trans*-orientation. *Pal*APP forms dimers 4.5-times more efficiently than total APP. Overexpression of the palmitoylating enzymes DHHC7 and DHHC21 that increase *pal*APP levels and Aβ release, also increased APP dimerization in cells. Conversely, inhibition of APP palmitoylation by pharmacological inhibitors reduced APP-dimerization in coimmunoprecipitation and FLIM/FRET assays. Finally, *in vitro* BACE1-activity assays demonstrate that palmitoylation-dependent dimerization of APP promotes β-cleavage of APP in lipid-rich detergent resistant cell membranes (DRMs), when compared to total APP. Most importantly, generation of sAPP_β_-sAPP_β_ dimers is dependent on APP-palmitoylation while total sAPP_β_ generation is not. Since BACE1 shows preference for *pal*APP dimers over total APP, *pal*APP dimers may serve as novel targets for effective β-cleavage inhibitors of APP as opposed to BACE1 inhibitors.

## Introduction

Amyloid precursor protein APP undergoes sequential proteolysis by β- and γ-secretases to generate amyloid β (Aβ). Deposition of the amyloid (Aβ) peptide in senile plaques is a hallmark of Alzheimer’s disease (AD) (*reviewed in* [1–3]). Shortly after synthesis in the ER, APP undergoes a number of post-translational modifications namely N- and O-glycosylation, acetylation and phosphorylation prior to trafficking to the Golgi and eventually to the plasma membrane. APP also undergoes novel lumenal palmitoylation in the ER where two cysteine residues, Cys^186^ and Cys^187^, incorporate 16-carbon palmitic acid to generate palmitoylated APP (*pal*APP) [4]. Approximately 10% of APP is palmitoylated *in vitro* and *in vivo* [4]. We, and others have reported that substituting palmitoylatable Cys^186^ or Cys^187^ with Ser/Ala significantly reduced Aβ generation *in vitro*, suggesting a role of APP palmitoylation in amyloidogenic processing of APP [4, 5].

The primary function of protein palmitoylation is to enhance hydrophobicity of proteins and target them to specific membrane compartments of the cell [6]. Protein palmitoylation is regulated by protein acyl transferases (PATs) that incorporate palmitic acid to proteins, and by de-palmitoylating protein thioesterases. Protein palmitoylation often targets proteins to the lipid raft microdomains [7, 8]. Protein acyl transferases (PATs) incorporate palmitic acid into proteins in a regulated manner, while de-palmitoylating protein thioesterases hydrolyze this bond. In the brain, protein palmitoylation is the most abundant lipid modification among neuronal proteins [9]. In addition to membrane localization, protein palmitoylation may also regulate protein-protein interactions and enhances homo- and hetero-dimerization of cellular proteins.

BACE1-inhibitors are promising therapeutic agents for AD treatment. Yet, no BACE1 inhibitor has been found effective in AD treatment. *pal*APP is enriched in the lipid rafts and undergoes BACE1-mediated β-cleavage [4]. Interestingly, raft-associated *pal*APP serves as a better substrate for BACE1 compared to total APP in cells and in mouse brains [4]. BACE1 and γ-secretase components also undergo palmitoylation. Similar to APP, palmitoylation of BACE1 and γ-secretase components targets these enzymes to lipid rafts [10–12]. Unfortunately, palmitoylation-deficient BACE1 or γ-secretase components did not alter APP processing *in vitro* [13, 14], although transgenic animals expressing palmitoylation-deficient γ-secretases (APH1 and nicastrin) showed reduced Aβ deposition via a yet unknown mechanism [15]. However, lipid-raft associated *pal*APP is a good substrate for β-cleavage and thus an optimal target for BACE1 inhibitors.

A large fraction (30%) of total membrane bound APP forms dimers, yet APP dimerization is a subject of controversy because its significance in APP function and/or processing is poorly understood [16]. APP homodimerization initiates in the ER [17], but APP dimers are also found in the Golgi and in the cell surface [18–20]. Likewise, APP palmitoylation is initiated in the ER, but *pal*APP is detected in lipid rafts, which are cholesterol-rich microdomains in Golgi and post-Golgi compartments [4]. APP dimerization is mediated by the extracellular domains E1 or E2, TM domain or by the Aβ containing conserved G^29^XXXG^33^ (numbers are based on Aβ numbering) domain (*Reviewed in* [21]). APP dimerization via the ectodomain (E1 and E2), in particular, appears to play significant role in APP processing [22]. Enforced dimerization of APP resulted in ~50% increase in Aβ production, while induced dimerization of APP C-terminal domain upon substitution of the glycine residues in the dimerization motif, GxxxG, reduced Aβ generation [23, 24].

Here, we report for the first time that APP palmitoylation in the E1-domain facilitates APP dimerization. A novel analysis combining palmitoylation- and dimerization-assays showed that *pal*APP forms ~4.5-fold stronger dimers compared to total APP. *Pal*APP-dimers are predominantly *cis*-oriented while *tot*APP dimerizes, as reported, in both *cis*- and *trans*-orientation. Mutants of APP exhibiting increased palmitoylation dimerized more efficiently than wild type APP. Coimmunoprecipitation and FLIM/FRET analyses using protein acyl transferases and/or palmitoylation inhibitors show that palmitoylation of APP modulates E1-mediated APP-dimerization. *In vitro* BACE1-activity assays revealed generation of sAPP_β_-sAPP_β_ dimers in lipid raft-containing detergent resistant membranes (DRMs), inhibited by palmitoylation inhibitors. Together, these findings demonstrate that APP-palmitoylation promotes APP-dimerization, and *pal*APP-dimers undergo β-cleavage in DRMs.

## Results

### APP palmitoylation promotes the formation of APP dimers

**Fig 1:**
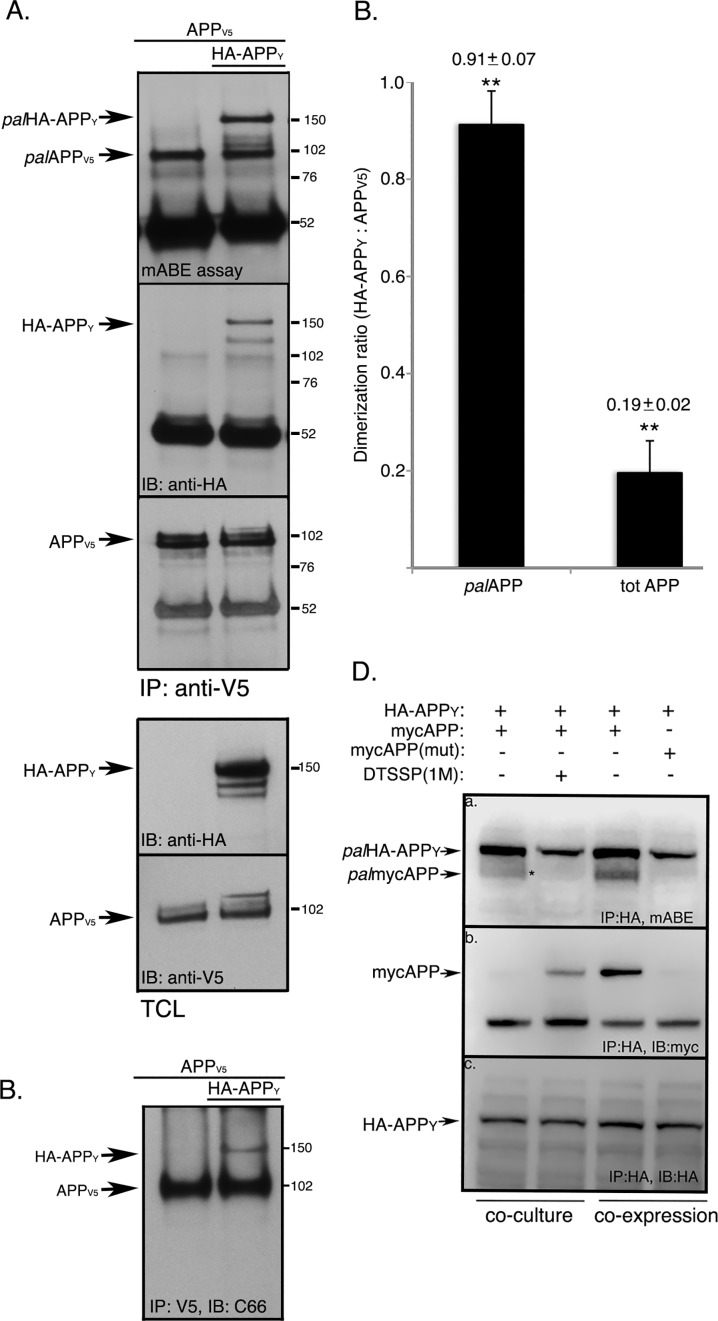
*pal*APP dimerizes ~4.5 times more efficiently compared totAPP and in cis-orientiation. A. Cells expressing APP_V5_ or APP_V5_ plus HA-APP_Y_ were subjected to co-immunoprecipitation assays to detect APP_V5_/HA-APP_Y_ interaction or APP-dimerization. APP_V5_ was immunoprecipitated with an anti-V5 antibody. Immunoprecipitates were probed with an anti-HA antibody to detect pull-down of HA-APP_Y_. Subsequently the immunoprecipitates were subjected to mABE assay to detect *pal*APP_V5_/HA-APP_Y_ interaction (or *pal*APP-dimerization). *Pal*APP_V5_ pulled down both *pal*APP_V5_ (M_wt_ ~102 kD) and *pal*HA-APP_Y_ (M_wt_ ~150 kD) from cells expressing APP_V5_ plus HA-APP_Y_ but not from cells expressing only APP_V5_. B. *Tot*APP-dimers (APP_V5_/HA-APP_Y_) only form in cells expressing both APP_V5_ and HA-APP_Y_. C. Quantitation of *pal*APP-dimers (*pal*APP_V5_/palHA-APP_Y_) versus *tot*APP-dimers (APP_V5_/HA-APP_Y_). Error bars show the s.e.m. (**p<0.01). D. *pal*APP dimerizes is *cis*-orientiation. Cells expressing HA-APP_Y_ and cells expressing mycAPP were co-cultured in absence or presence of 1mM cell-impermeable cross-linker DTSSP. Cell extracts were subjected to a pull-down assay, using an anti-HA antibody to immunoprecipitate HA-APP_Y_. To test for APP-dimerization, the precipitates were probed with an anti-myc antibody (*panel b*, *co-culture*). Cells co-expressing HA-APP_Y_ and mycAPP were also subjected to a co-IP assay using the anti-HA antibody to pull-down mycAPP with HA-APP_Y_.(*panel b*, *co-expression*). To detect *pal*APP-dimerization, the immunoprecipitates were also subjected to mABE assay to detect co-IP of *pal*HA-APP_Y_ with *pal*-mycAPP (*panel a*). The experiment is a representative of three independent experiments.

Several studies have shown that ectodomain-mediated APP dimerization requires hyodrophobic interactions, apart from the dimerization domains 18–350 and 448–465 in the N-terminus of the protein [19, 25]. Ectodomain-dependent dimerization of APP was shown to increase Aβ generation [23]. Since APP contains hydrophobic palmitic acid residues at its Cys^186^ and Cys^187^ in the ectodomain [4], we asked whether APP palmitoylation promotes APP dimerization. Here we performed an assay combining co-immunoprecipitation (co-IP) to assess dimerization and a modified acyl biotinylation assay (mABE) to assess palmitoylation of APP. For this assay, we used cells co-transfected with two expression plasmids. One expressed C-terminal V5-epitope tagged APP (APP_V5_), the other C-terminal YFP- and N-terminal HA-epitope tagged APP (HA-APP_Y_). As expected, HA-APP_Y_ efficiently co-immunoprecipitated with APP_V5_ (Fig 1A and 1B), confirming dimerization of APP. To test the presence of palmitoylated APP-dimers (*pal*APP_V5_-*pal*HA-APP_Y_), the precipitate was subjected to an mABE assay that not only detected ~100 kDa *pal*APP_V5_, but also identified the ~150 kDa *pal*HA-APP_Y_ (Fig 1A). This showed dimerization of *pal*APP_V5_ and *pal*HAAPP_Y_. Next we determined the stoichiometry of APP-APP and *pal*APP-*pal*APP interaction. For this we measured band intensities of pulled-down *pal*HA-APP_Y_ and that of *pal*APP_V5_. Quantitation revealed ~1:1 (0.91 ± 0.07) stoichiometry for *pal*HA-APP_Y_/*pal*APP_V5_ interaction (Fig 1C), suggesting that ~91% of *pal*APP formed dimers. We then compared the band intensities of immunoprecipitated *tot*HA-APP_Y_ and *tot*APP_V5_ (Fig 1B). Quantitation showed that *tot*HA-APP_Y_ coimmunoprecipitates with *tot*APP_V5_ with a modest 0.19 ± 0.02 stoichiometry (Fig 1C). These data demonstrate that *pal*APP undergoes near total (~91%) dimerization while only ~20% of *tot*APP forms dimers. Similar results were obtained when HA-APP_Y_ was immunoprecipitated prior to mABE assay. HA-APP_Y_ pulled down ~20% APP_V5_, while *pal* HA-APP_Y_ pulled down equal amount of *pal*APP_V5_ (data not shown). Our data provide evidence for the first time that palmitoylation of APP strongly promotes APP dimerization.

APP forms *cis-* and *trans-*dimers *in vitro* and *in vivo* [26, 27]. The cellular localization and function of APP may determine whether it dimerizes in *cis* or *trans* orientation [28]. Here we tested the orientation of *pal*APP dimers. We used co-IP assays on co-culture systems to ask whether *pal*APP is primarily dimerized in *cis* or *trans*. For this, cells expressing N-terminally myc-tagged APP (mycAPP) were co-cultured with cells expressing HA-APP_Y_. HA-APP_Y_ co-IPed mycAPP only in presence, but not in absence, of a cell impermeable cross-linker DTSSP at 4°C (Fig 1D, panel b, compare lanes 1 and 2). Given that the two proteins were expressed in different cell lines, this result indicates that mycAPP and HA-APP_Y_ dimerized in *trans-*orientation in presence of the cross-linker. Surprisingly, when we subjected the immunoprecipitates to mABE analysis, the same APP dimers in *trans* were found not to be palmitoylated (Fig 1D, panel a, lane 2). In contrast, HA-APP_Y_ not only pulled down mycAPP (Fig 1D, panel a, lane 3), but both HA-APP_Y_ and mycAPP were also palmitoylated (Fig 1D, panel b, lane 3), in experiments where HA-APP_Y_ and mycAPP were coexpressed in the same cell. A dimerization-defective mycAPP mutant containing the H108/110A mutation in the Growth Factor Like Domain (GFLD) of APP (mycAPP(mut)) showed little or no co-immunoprecipitation with HA-APP_Y_ (Fig 1D, panel a, lane 4) as expected from an earlier report [26]. Taken together, our data showed that *pal*APP did not form *trans*-dimers, thus suggesting *pal*APP-dimers were predominantly *cis-*oriented. Interestingly, *cis*-dimerization in particular is known to affect APP processing [22, 23, 29], increasing Aβ and sAPPβ generation [30].

**Fig 2:**
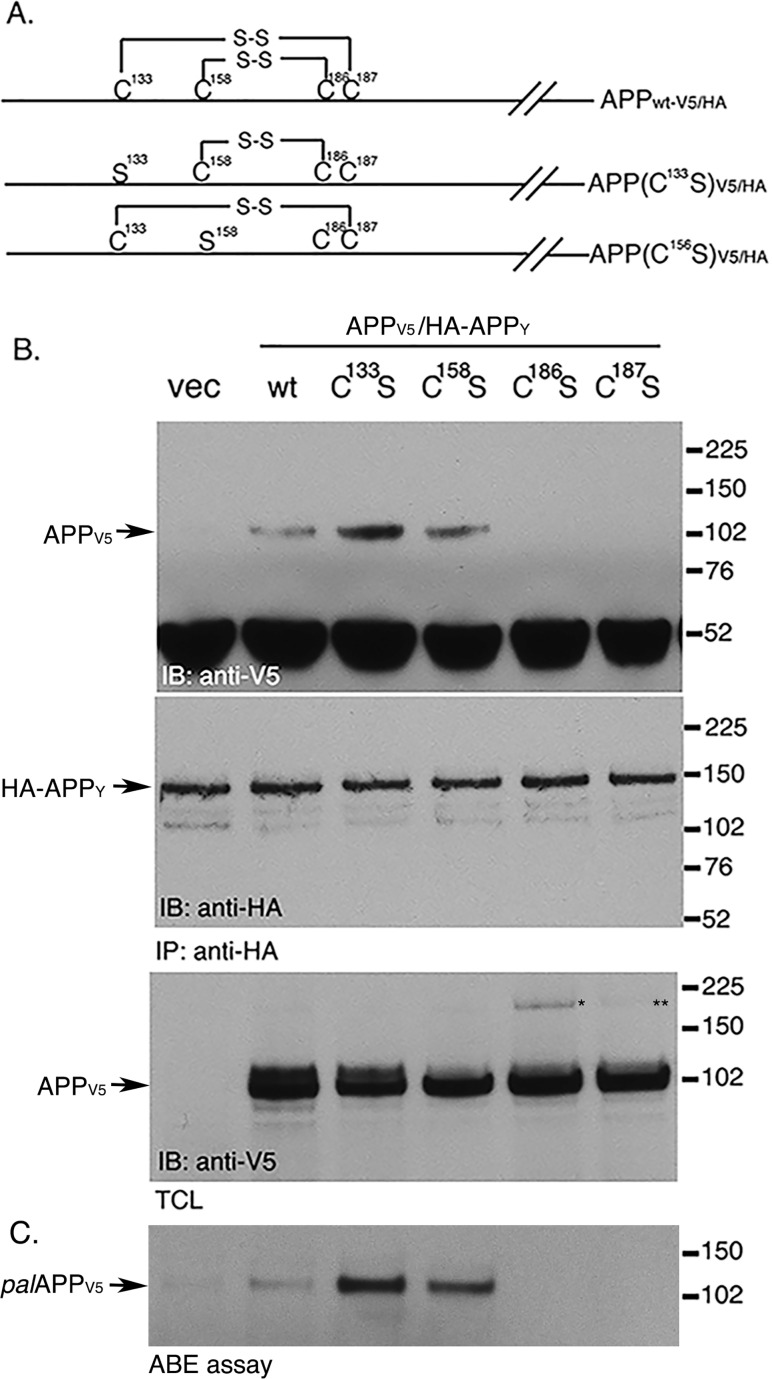
Palmitoylation-prone APP mutants exhibit increased APP dimerization compared to wtAPP. A. Schematic representation of the Cys to Ser mutants of APP used for the following co-immunoprecipitation assays. B. Co-immunoprecipitation assay in cells co-expressing APP_V5_ and HA-APP_Y_ and its mutants containing indicated Cys to Ser substitution. HA-APP_Y_ pulls down APP_V5_, indicating APP-APP dimerization. APP(C^133^S) and APP(C^158^S) show 2 fold increase in dimerization, while APP(C^186^S) and APP(C^187^S) fail to dimerize. APP(C^186^S) and APP(C^187^S) generated trace amounts of palmitoylation-independent dimers (* and **). C. ABE assay of cells overexpressing indicated APP mutants show 2 fold increased palmitoylation of APP(C^133^S) and APP(C^158^S), where as APP(C^186^S) and APP(C^187^S) were defective in palmitoylation.

To further confirm the direct correlation between APP palmitoylation and its dimerization, we have performed co-immunoprecipitation (co-IP) assays to test dimerization of APP in cells co-expressing HA- and V5-epitope-tagged palmitoylation-efficient APP mutants APP(C^133^S) and APP(C^158^S). We previously reported that substitution of Cys^133^ or Cys^158^ with serine (Ser) results in a ~2 fold increase in APP palmitoylation compared to APP_wt_, by freeing the palmitoylatable cysteins (Cys^186^ or Cys^187^) from forming disulfide (S-S) bridges with Cys^158^ and Cys^133^, respectively (Fig 2A, and [4]). Co-IP assays revealed that APP(C^133^S) and APP(C^158^S) form ~2 fold increased dimerization compared to that of APP_wt_ (Fig 2B), further confirming that increased *pal*APP levels increased APP-dimerization. In contrast, palmitoylation-deficient APP(C^186^S) and APP(C^187^S) showed little or no dimerization. Taken together, these results indicate that APP-palmitoylation promotes its dimerization.

### Palmitoyl acyl transferases DHHC7 and DHHC21, but not DHHC1, increase APP palmitoylation and dimerization

**Fig 3:**
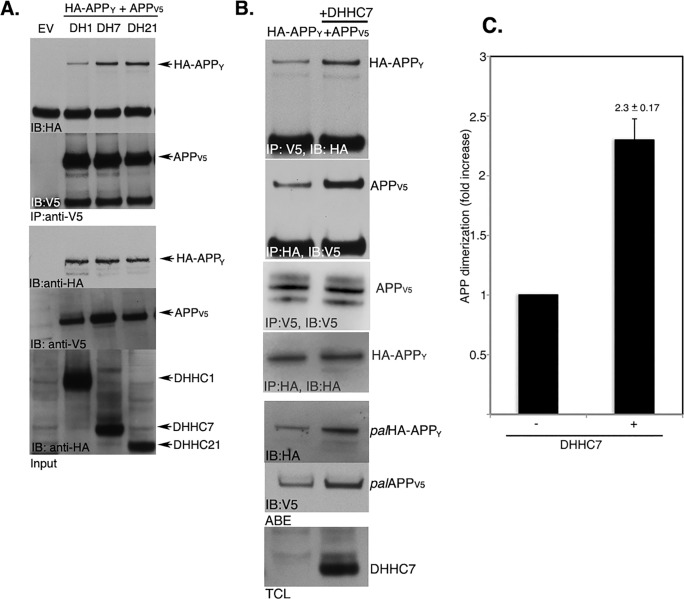
DHHC7 equally increases palmitoylation and dimerization of APP. A. Co-IP assays to detect dimerization of APP_V5_ and HA-APP_Y_ in presence or absence of indicated DHHC proteins. APP_V5_ pulled-down HA-APP_Y_, indicating APP dimerization. Overexpression of DHHC7 or DHHC21 increased co-IP of APP_V5_ and HA-APP_Y_, suggesting increased dimerization of APP in presence of these two palmitoylating enzymes. No effect on APP dimerization was observed in presence of DHHC1. EV represents empty vector. B. ABE analysis detected increased level of in *pal*APP (both *pal*HA-APP_Y_ and *pal*APP_V5_), as expected. C. Quantitation of dimerization assays (n = 3) detects 2.3 ± 0.17 fold increase of APP dimerization and ~2 fold (not shown) increase in APP palmitoylation in presence of DHHC7. Error bars show the s.e.m.

Because palmitoyl acyltransferases DHHC7 and DHHC21 consistently increased *pal*APP level without altering the amount of *tot*APP [4], we tested the effect of DHHC7 and DHHC21 on APP-dimerization. For this purpose, we used co-immunoprecipitation of HA-APP_Y_ with APP_V5_ in presence or absence of DHHC7 or DHHC21 to assess the effect of the DHHCs on APP dimerization (Fig 3). DHHC1 was selected as a negative control, as expression of DHHC1 does not promote APP palmitoylation [4]. DHHC7 and DHHC21 consistently increased co-IP of HA-APP_Y_ and APP_V5_, while DHHC1-overexpression had no effect on HA-APP_Y_/APP_V5_ coimmunoprecipitation (Fig 3A). Overexpression of DHHC7, in particular, not only increased APP dimerization (APP_V5_/HA-APP_Y_ interaction) (Fig 3B), but also consistently increased *pal*APP_V5_ and *pal*HA-APP_Y_ levels in our ABE analysis (Fig 3B). Quantitation of APP dimerization revealed that DHHC7 increased APP dimerization by 2.3 ± 0.17 fold (Fig 3C). DHHC7 also increased *pal*APP_V5_ and *pal*HA-APP_Y_ levels by ~2 fold (Fig 3B), as expected. Thus, our data show a direct correlation between APP palmitoylation and APP dimerization.

### Palmitoylation inhibitors reduce APP dimerization

**Fig 4:**
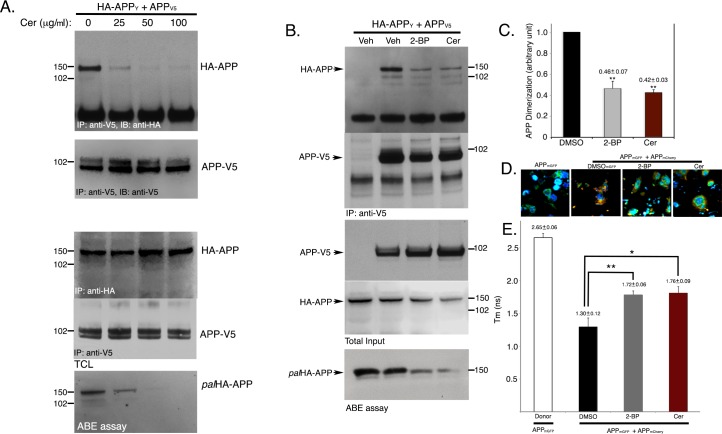
Palmitoylation inhibitors impair APP dimerization in CHO cells. A. Co-immunoprecipitation assay of CHO cells expressing HA-APP_Y_ and APP_V5_ in presence of increasing amounts of cerulenin (Cer) (0–100 μg/ml), where 0 μg/ml represents DMSO-treatment. Cerulenin decreased HA-APP_Y_/APP_V5_ interaction (APP-dimerization) in dose dependent manner similar to cerulenin’s effect on APP-palmitoylation. B. 25 μg/ml cerulenin and 50 μM 2-BP reduced both APP-palmitoylation and APP-dimerization in HA-APP_Y_/APP_V5_-expressing cells compared to DMSO (Veh)-treated cells. C. Quantitation showed 54 and 58% decrease of APP-APP dimerization by cerulenin (25 μg/ml) and 2-BP (50 μM), respectively. D. Naïve CHO cells were either transiently transfected with an expression plasmid encoding APP_mGFP_ or co-transfected with expression plasmids encoding APP_mGFP_ and APP_mCherry_. After 24 h, cells were either treated with DMSO or with indicated palmitoylation inhibitors cerulenin or 2-BP for 6 h prior to formalin treatment. FRET/FLIM analysis was employed to measure decay time constant T_m_ of APP_mEGFP_ in cells expressing APP_mEGFP_ (n = 54), and APP_mGFP_ and APP_mCherry_ (n = 46). T_m_ of life-time decay of APP_mEGFP_ in APP_mEGFP_/APP_mCherry_ co-expressing cells was monitored in absence and in presence of palmitoylation inhibitors (25 μg/ml cerulenin or 50 μM 2-BP). Quantitation revealed that inhibitors increased T_m_ values by ~1.5 fold, indicating disruption of dimerization between APP_mGFP_ and APP_mCherry_.

Next we asked whether inhibition of APP-palmitoylation affects APP-dimerization. Here, we tested the effect of two palmitoylation inhibitors, 2-bromopalmitate (2-BP) and cerulenin, on APP-dimerization because they had induced robust decrease of APP palmitoylation in our earlier report. Cells co-expressing HA-APP_Y_ and APP_V5_ were subjected to cerulenin treatment prior to co-IP assay. Co-immunoprecipitation of HA-APP_Y_ and APP_V5_ was decreased by cerulenin-treatment in a dose dependent manner (Fig 4A). In a separate experiment, cerulenin also decreased generation of Aβ_40_ and Aβ_42_ in APP-expressing cells (CHO_APP_) in a dose-dependent manner. Specifically, conditioned media from CHO_APP_ cells generated 331.8±14.5 and 12.4±0.6 pmol/L Aβ_40_ and Aβ_42_, respectively. 25, 50 and 100 μg/ml cerulenin treatment reduced Aβ_40_ levels to 239.4±39.4, 145.6±13.9 and 126.5±17.7 pmol/L, respectively. Aβ_42_ level was reduced to 7.7±0.4, 5.6±0.4 and 4.6±0.2 pmol/L, respectively (S1 Fig). Thus, inhibition of APP palmitoylation not only leads to disruption of APP-dimerization, but also reduces Aβ generation. Similar to cerulenin, 2-BP also dramatically reduced HA-APP_Y_/APP_V5_ co-immunoprecipitation (Fig 4B). Quantitation showed ~56% reduction of HA-APP_Y_/APP_V5_ co-immunoprecipitation by 50 μM 2-BP, while 25 μg/ml cerulenin -treatment reduced the interaction by nearly 58% (Fig 4C). As expected, cerulenin and 2-BP also caused a 50% reduction in palmitoylated APP (*pal*HA-APP_Y_ and *pal*APP_V5_) levels without affecting total HA-APP_Y_ and APP_V5_ levels. Our data showed that inhibition of APP palmitoylation reduced APP dimerization, further confirming a correlation between these two modifications.

To validate the effect of 2-BP and cerulenin on APP dimerization we determined the 2pFLIM efficiency of two interacting APP molecules in absence or presence of the inhibitors. For this purpose we transfected cells with APP C-terminally tagged with mGFP (APP_mGFP_) and mCherry (APP_mCherry_). FRET measurements were taken by using APP_mGFP_ as donor and APP_mCherry_ as acceptor, as described by Fogel, H. *et al* (Fig 4D). Briefly, the 2pFLIM method is based on the fact that that shortening of donor lifetime indicates FRET. APP_mEGFP_ alone showed lifetime decay, displaying a time constant T_m_ of 2.65 ± 0.06 ns (Fig 4E). FRET between APP_mEGFP_ and APP_mCherry_ decreased the T_m_ to 1.3 ± 0.02 ns (Fig 4E), indicating a strong APP_mEGFP_-APP_mCherry_ interaction. 2-BP (50 μM) and cerulenin (25 μg/ml) treatment brought up the time constant to 1.76 ± 0.06 and 1.72 ± 0.09 (Fig 4E), respectively, as these compounds reduced APP_mEGFP_- APP_mCherry_ interaction. FRET analysis revealed a ~32 and a ~35% reduction in APP dimerization by 2-BP and cerulenin, respectively. Here, we further demonstrated that reduction in *pal*APP levels by palmitoylation-inhibitors (cerulenin and 2-BP) reduced APP dimerization.

So far, we confirmed that palmitoylation inhibitors decreased APP dimerization. While co-IP assays and FRET/FLIM analyses yielded the same result, the decrease in APP dimerization was more pronounced in our co-IP assays whereas FRET assays did not completely correlate with decrease in APP palmitoylation and dimerization. Specifically, co-IP assays showed that 25 μg/ml cerulenin and 50 μM 2-BP reduced both APP-palmitoylation and APP-dimerization by 50%. FRET/FLIM analyses only yielded a consistent ~33% in APP-dimerization by the same inhibitors that reduced *pal*APP level by ~50%.

**Fig 5:**
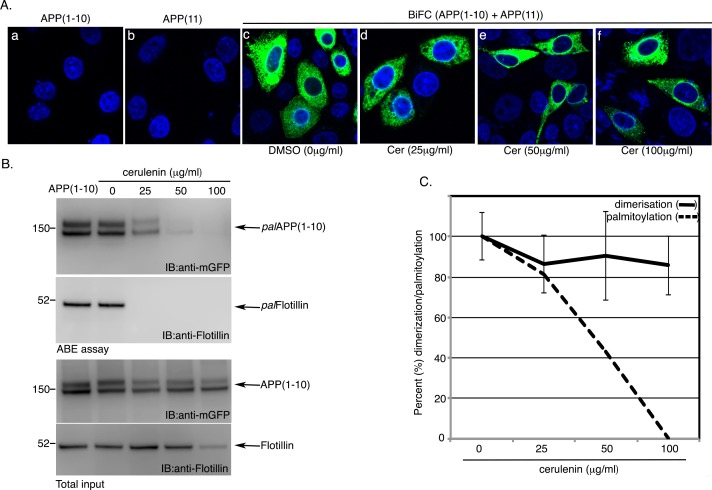
Bimolecular Fluorescence Complementation (BiFC) assay detects APP dimers. A. Fluorescence microscopy of cells transiently expressing APP C-terminal Split GFP 1–10 (APP(1–10)) and APP C-terminal split GFP 11 (APP(11)), FACS sorted for equal intensity. Cells showing green fluorescence represents the APP dimers. These cells were sorted in a fluorescence activated cell sorter (FACS) to obtain equal intensity cells. Next the sorted cells were grown on coverslips for 18 h before treating with 0 (DMSO), 25, 50 and 100μg/ml cerulenin for 6 h showed little or no change in fluorescence intensities. B. Cells expressing APP(1–10) or APP(1–10)+APP(11) were subjected to ABE assay. Probing the samples with anti-GFP detected palmitoylaed APP(1–10) (*pal*APP(1–10)). Anti-Flotillin antibody detected palmitoylated flotillin (*pal*Flotillin) in ABE assay. C. BiFC intensities of the cells were quantitated using ImageJ software. Intensities of more than 50 cells were measured for each treatment. Average intensities are plotted in percent (%) change in dimerization, using no treatment as 100% describing changes in APP-dimerization percent by increasing amount of cerulenin (*solid line*). The *discontinuous line* represents decrease in *pal*APP levels obtained from ABE analysis upon cerulenin treatment. Error bars show the s.e.m.

To further confirm the effect of palmitoylation inhibitors on APP-dimerization we attempted another approach. We employed bimolecular fluorescence complementation (BiFC) assays on cells co-expressing two APP constructs, APP-GFP(1–10) and APP-GFP(11), containing split-GFP. BiFC assays were performed as described by Isbert *et al*. [17]. APP-GFP(1–10) and APP-GFP(11) expression plasmids contained two non-fluorescence portions of GFP fused separately to the N-terminus of APP. GFP’s full fluorescence property, called BiFC, is restored when APP-GFP(1–10) and APP-GFP(11) are brought together due to APP-APP association. Here, we co-expressed expression plasmids encoding APP-GFP(1–10) and APP-GFP(11) in naïve CHO cells. Cells expressing APP-GFP(1–10) or APP-GFP(11) did not generate any fluorescence (Fig 5A, *a* and *b*, respectively), as expected, while cells co-expressing the split-GFP plasmids, APP-GFP(1–10) and APP-GFP(11), produced robust fluorescence (Fig 5A, c) as shown before [17], suggesting APP-APP dimerization. The co-expressing cells were then sorted by a fluorescence-activated cell sorter (FACS) to obtain homogenous cultures of cells expressing APP-GFP(1–10) and APP-GFP(11). FACS sorted cells co-expressing APP-GFP(1–10) and APP-GFP(11) were grown on coverslips over night before treating with increasing amounts of cerulenin (0–100μg/ml) for 3 h (Fig 5A, c-f). Vehicle (DMSO)-treated cells (0 μg/ml) exhibited fluorescent signal (Fig 5A, c) as expected. Surprisingly, cells treated with either 25, 50 or 100μg/ml cerulenin showed little or no change in fluorescence intensities (Fig 5A, *d*, *e* and *f*, respectively), although 25, 50 and 100μg/ml cerulenin-treatment decreased *pal*APP(1–10) levels by ~19, ~57 and ~99%, respectively (Fig 5B and 5C). It was surprising that the effect of APP-dimerization by palmitoylation-inhibitors in our BiFC analysis did not yield even 33% decrease that was observed in our FLIM/FRET analysis. It is possible that BiFC APP and untagged APP differ in their stability, thus masking the effect of the palmitoylation inhibitors. Constitutively expressed BiFC APP (APP(1–10)) and untagged APP (APP) showed half-lives of 2–3 h (S2A Fig) similar to earlier reports describing the half-life of APP as ~4 h [31]. Thus, the little or no effect of cerulenin on BiFC APP dimerization is not due to stronger stability of the BiFC APP mutants. We also tested the half-life of *pal*APP by pulse chase analysis where CHO_APP_ cells were first labeled with chemically reactive palmitic acid, Alkyl-C16, as before [4] followed by chasing with unlabeled palmitic acid for 0.5, 1, 3 and 6 h. *pal*APP was detected after labeling Alkyl-C16 incorporated APP with fluorescent TAMRA using Click-iT technique as before [4]. Half-life of *pal*APP appeared to be 3–6 h (S2B Fig), suggesting no change in the stability of *pal*APP compared to *total*APP. Although the data does not indicate whether *pal*APP dimers are more stable than non-*pal*APP, it will be interesting to determine the half-life of dimerized *pal*APP in future when and if an antibody specific for *pal*APP becomes available.

**Fig 6:**
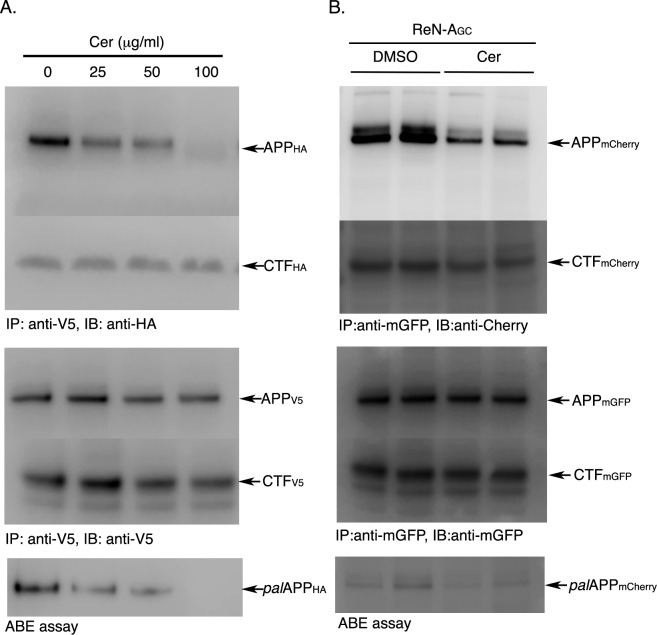
Palmitoylation inhibitors specifically impair ectodomain-dependent APP dimerization but not APP-CTF-dimerization. A. Naïve CHO cells co-expressing APP_V5_ and APP_HA_ were subjected to a co-IP assay in presence of DMSO (0 μg/ml) or increasing concentrations of cerulenin (25, 50 and 100 μg/ml). *fl*APP_V5_ (APP_V5_) pulled down *fl-* as well as the C-terminal fragments of APP_HA_ (APP_HA_ and CTF_HA_, respectively) in DMSO-treated (0 μg/ml cerulenin) cells. In presence of cerulenin, co-IP of *fl*APP_V5_ (APP_V5_) with *fl*APP_HA_ (APP_HA_) decreased in a dose-dependent manner. Little or no co-IP of *fl*APP observed upon treatment with100 μg/ml cerulenin. In contrast, cerulenin had no effect on CTF_HA_ pull-down even at the highest concentration (100 μg/ml). Cerulenin reduced *pal*APP_HA_ levels in a dose-dependent manner (ABE assay) reaching complete inhibition at 100 μg/ml concentration. B. co-IP assay using an antibody specific for mGFP (anti-mGFP) to pull-down full-length (fl) APP_mGFP_ with APP_mCherry_ from differentiated neuronal cells (RenVM) co-expressing APP_mGFP_+APP_mCherry_. Anti-mGFP also pulled-down CTF_mCherry_ with CTF_mGFP_. Cerulenin (25 μg/ml) treatment of the cells prior to co-IP assay dramatically decreased *fl*APP_mGFP_-*fl*APP_mCherry_ interaction, but not that of CTF_mGFP_-CTF_mCherry_.

We now asked if dimerization of APP C-terminal fragments (CTFs), only detected in our FRET/FLIM or BiFC analyses, in addition of full-length APP, could explain the discrepancy between the co-IP analysis and the fluorescence-based methods. To distinguish between full-length APP and APP-CTF dimerization, we tested the effect of the palmitoylation inhibitors on APP-APP and CTF-CTF interactions in cells expressing C-terminally V5- or HA-epitope tagged APP (APP_V5_ and APP_HA_, respectively) (Fig 6A). Again, we detected CTF_V5_/CTF_HA_-dimerization in addition to full-lenghth APP_V5_/APP_HA_- dimerization (Fig 6A). Similar to cells co-expressing APP_mGFP_ and APP_mCherry_, cerulenin (25 μg/ml) and 2-BP (50 μM) reduced APP_V5_/APP_HA_-dimerization by ~50% without affecting CTF_V5_/CTF_HA_-dimerization when compared to total APP (*tot*APP) or total CTF (*tot*CTF) levels (Fig 6A). As expected, cerulenin (25 μg/ml) and 2-BP (50 μM) reduced both *pal*APP_V5_ and *pal*APP_HA_ levels by ~50%. Most importantly, 100 μg/ml cerulenin not only completely reduced *pal*APP level, but also reduced APP-dimerization to similar extent without affecting CTF-dimerization (Fig 6A). The data demonstrate that reduction in *pal*APP levels by palmitoylation inhibitors specifically reduced ectodomain-mediated dimerization of APP but has no effect on CTF-CTF dimerization. As APP is palmitoylated in its ectodomain, our results strongly indicate that palmitoylation of APP in its ectodomain regulates ectodomain-mediated APP-APP dimerization.

Next we tested the effect of palmitoylation inhibitors on APP-dimerization in human neural stem cells (ReN cells, Millipore) differentiated into mature neurons. Differentiated Ren lines containing FAD mutants inside a 3-D matrix has been demonstrated as a potential cellular model for AD [32]. Here, we infected naïve ReN-VM cells with lentiviral particles containing expression vectors for APP_mGFP_ or APP_mCherry_. Cells were then sorted by a fluorescence-activated cell sorter (FACS) to obtain homogenous cultures of cells expressing APP_mGFP_ (ReN-A_G_) or APP_mGFP_+APP_mCherry_ (ReN-A_GC_) (S3A Fig). The sorted cells were allowed to differentiate into neurons as described by D’Avanzo *et al*. [33] (S3B Fig) prior to co-IP analysis in absence or presence of palmitoylation inhibitors. A pull-down assay using an antibody specific for the mGFP (anti-GFP) epitope co-immunoprecipitated APP_mGFP_ and APP_mCherry_, suggesting APP_mGFP_-APP_mCherry_ interaction (Fig 6B). Interestingly, the C-terminal fragment of APP_mGFP_ (CTF_mGFP_) also co-precipitated with the CTF of APP_mCherry_ (CTF_mCherry_), suggesting CTF_mGFP_-CTF_mCherry_ interaction. Cerulenin (25 μg/ml) and 2-BP (50 μM) reduced APP_mGFP_-APP_mCherry_ interaction by ~50%. In contrast, cerulenin or 2-BP showed little or no effect on CTF_mGFP_-CTF_mCherry_ interaction (Fig 6A), further confirming that inhibition of APP palmitoylation prevents full length APP-APP dimerization without affecting CTF-CTF dimerization.

### *pal*APP dimers serve as substrates for β-cleavage in cell membranes

The APP ectodomain (E1) regulates a number of APP-functions, such as synaptogenesis [26] or glutamate release via the G_i/o_-signaling pathway [27]. Induced dimerization of APP via its E1 domain increased Aβ production and sAPPβ release [23, 24]. Since *pal*APP is a better substrate for β-cleavage compared to total APP, we asked whether *pal*APP dimers undergo β-cleavage. We hypothesized that dimerized *pal*APP would produce dimerized *pal-*sAPPβ after proteolysis by BACE1. First, we attempted to identify sAPP dimers in the conditioned media of cells co-expressing N-terminally myc-epitope tagged APP (mycAPP) and HA-APP_Y_. Both myc-sAPP and HA-sAPP were detected in the conditioned media, but we were unable to detect any dimerized sAPP in the CM via co-IP assays (*data not shown*). We then reasoned that *pal*APP dimers, upon proteolysis by β-secretase, may produce *pal-*sAPPβ dimers anchored to cell membranes. However, we again failed to detect *pal-*sAPPβ dimers in total or lipid raft membranes (*data not shown*). Although this observation did not entirely eliminate the possibility of *pal*-sAPP dimers in the conditioned media, it appears that their presence may be below our detection limit. Developing a *pal*-APP-specific antibody will be necessary to detect *pal*-sAPP dimers in the conditioned media.

**Fig 7:**
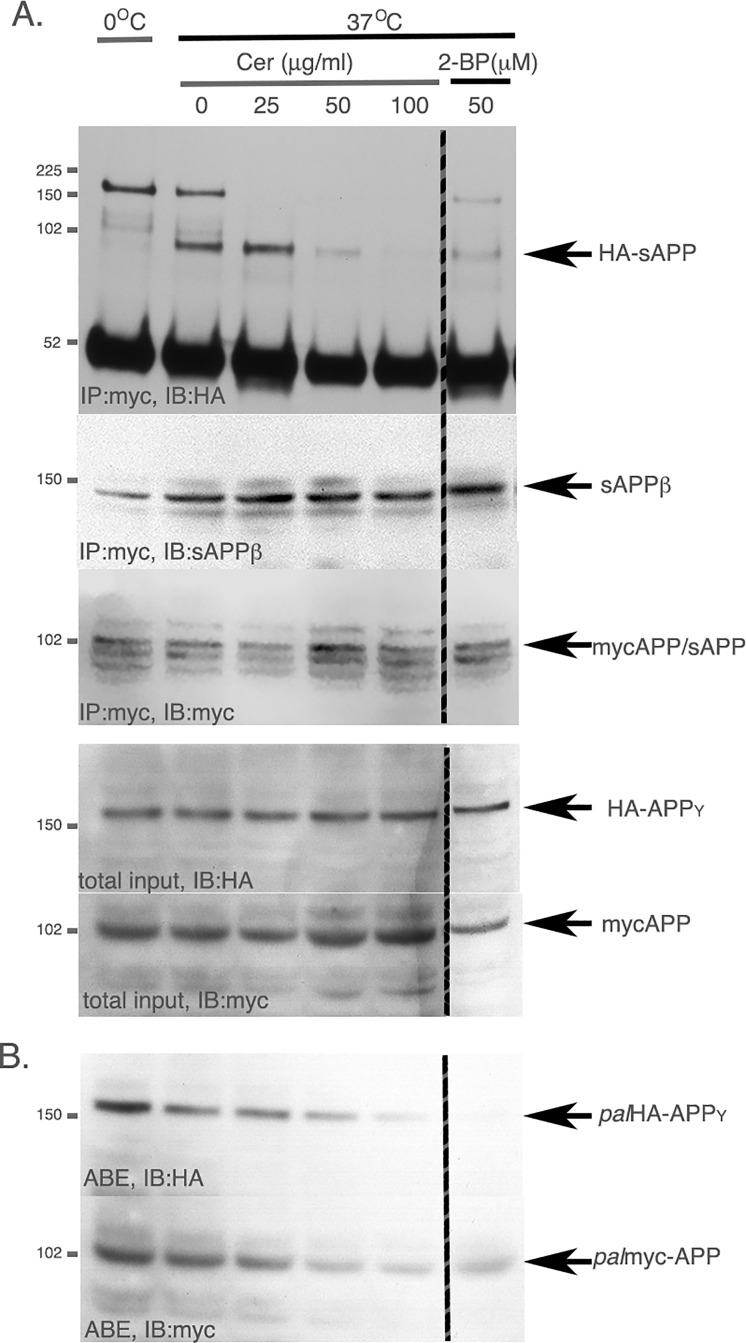
In vitro BACE-activity assay on palAPP-dimers in detergent resistant membranes (DRMs). A. *In vitro* BACE-activity assay on DRMs isolated from CHO cells expressing HA-APP_Y_ and myc-APP, treated with increasing amounts of cerulenin (Cer) prior to membrane preparation. The *in vitro* BACE-activity assay was followed by co-IP analysis as described in *Materials and Methods*. Myc-sAPP_β_ pulled down HA-sAPP_β_ in absence of cerulenin. In presence of cerulenin, pull-down of HA-sAPP_β_ with myc-sAPP_β_ decreased in a dose dependent manner. 2-bromopalmitate (100μM) dramatically reduced co-IP of HA-sAPP_β_ and myc-sAPP_β_. *B*. ABE assay of cells co-expressing HA-APP_Y_ and myc-APP. *pal* HA-APP_Y_ and *pal*myc-APP decreased upon cerulenin-treatment in a dose-dependent manner. C. Representation of dose-dependent decrease in HA-sAPP_β_ pull-down with myc-sAPP_β_ in presence of cerulenin.

To increase the sensitivity of our assay, we turned to *in vitro* experiments. We previously reported that *pal*APP is a better substrate than *tot*APP for BACE1-mediated β-cleavage in *in vitro* studies, using detergent resistant lipid raft microdomains. Thus, we next asked whether *pal*APP dimers are better substrates than *tot*APP for β-cleavage in an *in vitro* BACE-activity assay in detergent resistant membranes (DRM). DRMs were rich in lipid rafts as evident from enriched amounts of raft-resident protein flotillin in these membrane fractions compared to that in non-DRM fractions (*data not shown*). DRMs also showed the presence of high levels (~20%) of *pal*APP compared to that in non-DRM fractions. To detect the generation of sAPPβ dimers in these *pal*APP-rich DRMs, we performed co-IP experiments after *in vitro* BACE1-activity assays of DRMs isolated from HA-APP_Y_/mycAPP-expressing (Fig 7A). To stabilize released *pal*HA-sAPP_β_ /*pal*myc-sAPP_β_ dimers, we pretreated HA-APP_Y_ and mycAPP coexpressing cells with the mild crosslinker DSS (disuccinimidyl suberate) at a low concentration (50 μM) as described by Fogel, H *et al*. [27] prior to DRM preparation. DRMs isolated from these cells were incubated in acetate buffer, pH 4 at 37°C for 1h to generate sAPP_β_. The membranes were then subjected to co-IP analysis where myc-sAPP_β_ generated upon β-cleavage of the mycAPP/HA-APP_Y_-dimer were precipitated using an anti-myc antibody to pull-down HA-sAPP_β_ (Fig 7A). Interestingly, the anti-myc antibody not only pulled down ~100 kD HA-sAPP to confirm myc-sAPP/HA-sAPP interaction, but also co-precipitated ~150 kD *fl*HA-APP_Y_ indicating the presence of residual myc- APP/HA-APP_Y_ dimers in the assay (Fig 7A, *upper panel*). Most importantly, an anti-sAPP_β_ antibody stained a pulled-down ~100kD sAPP (Fig 7A, *IB*:*anti-sAPP*β), indicating that the myc antibody pulled-down HA-sAPP (Fig 7A, *upper panel*) as sAPP_β_. This showed that myc-sAPP_β_/HA-sAPP_β_ dimers were present after β-cleavage of APP-APP (myc-sAPP/HA-sAPP_Y_) dimers.

Pretreatment of the cells with the palmitoylation inhibitor cerulenin prior to BACE1- activity assay revealed dramatic reduction of the HA-sAPP_β_ pull-down with myc-sAPP_β_ in a dose dependent manner (Fig 7A, *upper panel*), demonstrating palmitoylation-dependent release of sAPP_β_-dimers. Importantly, cerulenin showed virtually no effect on total sAPPβ release as we observed little or no reduction in *tot-*sAPPβ levels (Fig 7A, *IB*:*sAPP*β). Notably, cerulenin decreased both *pal*HA-APP_Y_ and *pal*myc-APP levels in a dose-dependent manner (Fig 7B). Similar results were obtained when the cells were pretreated with the palmitoylation inhibitor 2-bromopalmitate (Fig 7A and 7B, *2-BP lanes*). Together, our observations revealed that APP dimers (HA-APP_Y_/mycAPP-dimer) released sAPP_β_-dimers (HA-sAPP_β_/myc-sAPP_β_-dimer) in a palmitoylation-dependent manner, while *tot*-sAPPβ release was independent on protein palmitoylation. This observation indicates that *pal*APP dimers are a better substrate for BACE1 cleavage compared to *tot*APP in DRMs.

## Discussion

We previously reported that *pal*APP serves as a better BACE1-substrate than *tot*APP. Here we show that over 90% of *pal*APP is found in dimers and that *pal*APP dimers are 4.5-times more enriched than *tot*APP dimers. BACE1 cleaves *pal*APP dimers more efficiently than *tot*APP. This finding may prove to be important for the design of effective β-cleavage inhibitors of APP, as opposed to BACE1 inhibitors.

APP forms homodimers and higher-order oligomers in heterologous expression systems and in brain homogenates [23, 34, 35]. Dimerization via the GXXXG motif alters γ-secretase activity [24] and plays an important role in the processing of Aβ40/Aβ42 into shorter Aβ species [16]. APP dimerization through the GXXXC-motif had no effect on its BACE-cleavage, but loss of dimerization via GXXXC has been shown to inhibit the production of Aβ_42_ [24]. APP dimerization via the ectodomain (E1 and E2), in contrast, appears to play significant role in APP processing [22]. E1-mediated APP dimerization has been reported to mediate APP’s synaptogenic functions [26]. E1-mediated dimerization of APP has also been shown to induce APP-APP conformational changes and presynaptic enhancement, leading to Aβ40-mediated APP/Gi/o-induced glutamate release [27]. Forced dimerization of APP increased Aβ production, while inducing dimerization of APP C-terminal domain via mutation of the transmembrane GxxxG motif reduced Aβ generation [23, 24]. The ectodomain-mediated APP-dimerization appeared to play a more significant role in AD pathophysiology than its C-terminal mediated dimerization. Yet, APP-dimerization via its ectodomain is controversial because purified or overexpressed APP-ectodomain(s) are primarily monomeric, and can only form dimers at very high concentrations or in presence of heparin [22, 36, 37]. Although recent reports show that the GFLD (Growth factor like domain) and CuBD (Copper binding domain) of APP are essential for APP-dimerization [26], GFLD generated as a stable protease-resistant degradation product of the APP ectodomain (18–350) is monomeric [25]. We were also unable to detect dimeric sAPP in the conditioned media of APP-overexpressing cells, as expected from earlier reports [22, 36, 37]. It has been predicted earlier that ectodomain-mediated APP-dimerization requires unknown hydrophobic interactions [35]. Since post-translational palmitoylation provides hydrophobicity for protein-lipid and protein-protein interactions, our discovery that *pal*APP forms dimers 4.5-times more efficiently than *tot*APP is consistent with the earlier report.

An interesting question is whether APP-palmitoylation directly mediates its dimerization. APP-dimerization initiates in the ER [19]. Palmitoylation-deficient APP mutants (APP(C^186^S) and APP(C^187^S)) show little or no dimerization and are retained in the ER [4]. It is worth noting that we often detect a ~200 kD band (Fig 2B, *) appearing from APP(C^186^S) mutant. APP(C^187^S) also generates similar band to much lesser extent (Fig 2B, **). Since both APP(C^186^S) and APP(C^187^S) mutants are predominantly ER-bound [4], we speculate that the bands are APP-dimers because ER-targeted APP (ER-APP) has been reported to generate a strong ~200 kD band indicating APP-dimerization initiating in the ER [17]. In addition to strong ER-binding these mutants also lack palmitoylation. Thus the trace amount of APP-dimers from these mutants may be palmitoylation-independent APP-dimers. Increased palmitoylation of APP, either by APP(C^133^S) and APP(C^158^S) mutations or by overexpression of the palmitoylating enzyme DHHC7 that can increase *pal*APP levels also show concomitant increase in APP-dimer levels (Figs 2 and 3). In contrast, co-IP, FRET/FLIM and BiFC analyses of cells treated with pharmacological inhibitors of palmitoylation showed robust (in co-IP assay) or moderate (FRET/FLIM and BiFC assays) decrease in APP dimerization (Figs 4 and 5). The palmitoylation inhibitors cerulenin not only reduced *pal*APP levels, but also inhibited APP-APP dimerization in a dose-dependent manner (Fig 4A). Moreover, palmitoylation-inhibitors specifically reduced *fl*APP-dimerization without affecting CTF-dimerization (Fig 6). Since palmitoyl moieties are incorporated in the ectodomain of APP, reduction of APP-palmitoylation and APP-dimerization by these inhibitors strongly suggests a direct effect of APP-palmitoylation on its dimerization. However, the significance of other dimerization sites such as the growth-factor-like domain (GFLD), the copper binding domain (CuBD) or the E1 (91–111) region in APP-dimerization cannot be ruled out. We may have uncovered a series of sequential events initiating with APP-palmitoylation, that promotes its ectodomain-mediated dimerization. Further studies will be required to verify this hypothesis. Since APP-dimers show differential susceptibility towards external stimuli based on the subcellular localization of the dimers [27], we predict that APP requires palmitoylation domains and/or additional domain(s) for dimerization in a spatial and temporal manner.

The role of ectodomain-mediated dimerization of APP in APP processing is still under investigation. Palmitoylation inhibitors not only abrogate APP-APP interaction, but also reduce APP-CTF_α/β_ generation and Aβ production in cells [4]. However, our co-IP experiments failed to directly detect HA-sAPP_β_ /myc-sAPP_β_ -dimers in the conditioned media of cells co-expressing HA-APP_Y_ and mycAPP (*data not shown*). We were also unable to detect HA-*pal*sAPP_β_ /myc-*pal*sAPP_β_ -dimers in the conditioned media. Although high levels of both myc- and HA-sAPP were released in the conditioned media (*data not shown*), our failure to detect both total and palmitoylated sAPP-dimers is not surprising because purified ectodomain of APP is primarily monomeric in solution [22, 37]. We have reported earlier that *pal*APP is targeted to the detergent resistant cholesterol rich microdomains called lipid rafts. We have also shown that raft-associated *pal*APP serve as a better BACE1-substrate compared to *tot*APP [4]. Thus, we reasoned that *pal*sAPP_β_ dimers could be embedded in lipid-rich membranes because of the hydrophobic nature of palmitoyl-moiety. Consistently, in presence of a mild cross-linker we detected sAPP_β_-dimers in detergent resistant membranes (DRMs) rich in lipid rafts. Importantly, release of sAPP_β_-dimers, but not that of *tot*-sAPP_β_, was susceptible to the palmitoylation inhibitors cerulenin and 2-BP (Fig 7). Generation of sAPP_β_-dimers was decreased by cerulenin in a dose-dependent manner corresponding to the decrease in *pal*APP levels (Fig 7A and 7B). Decrease of sAPP_β_-dimer formation in presence of palmitoylation inhibitors suggests that sAPP_β_-dimers were formed from *pal*-APP dimers in DRMs. A direct detection of *pal*sAPP_β_ dimers *in vivo* is necessary for further studies on the role of *pal*APP dimerization in APP processing.

BACE1-mediated β-cleavage of APP is the rate-limiting step for Aβ generation. Unfortunately, development of BACE1 inhibitors for AD treatment is difficult due to the promiscuity of BACE1 for its substrates. Moreover, APP is primarily a substrate for α-cleavage producing non-amyloidogenic peptides. Relocalization of APP into cholesterol-rich lipid rafts shifts APP towards amyloidogenic cleavage by BACE1 (*reviewed in* [38]). We have reported that *pal*APP is enriched in lipid rafts favoring β- over α-cleavage [4]. We also reported that lipid raft-associated *pal*APP is a better substrate *in vitro* and *in vivo*. Now we show that *pal*APP forms stronger dimers than *tot*APP primarily in *cis*-orientation (Fig 1D), which is considered more favorable dimer orientation for β-cleavage. Although, a direct proof that BACE1 cleaves *pal*APP-dimers more efficiently than non-*pal*APP requires further investigation, our data strongly indicate that *pal*APP-dimers are better substrates for β-cleavage in DRMs than *tot*APP. It is encouraging that a recent High Throughput Screen of nearly 77,000 compounds identified two small molecule modulators of *tot*APP dimerization that may lower sAPP_β_ levels without affecting α- or γ-cleavage [39]. Our data indicate that small molecules designed to specifically reduce *pal*APP-dimer formation would be potent inhibitors of APP’s β-cleavage in the brains of patients affected by AD.

Our finding that APP-palmitoylation is a novel contributor to APP-dimerization adds the palmitoylated cysteines (Cys^186^ and Cys^187^) to the previously identified multiple dimerization interfaces in APP. The multi-fasceted dimerization of APP provides a potential for different conformations of the protein, leading to different effects on β-cleavage and Aβ generation. Thus, the dimerization domain plays an essential role in predicting whether APP-dimers increase or decrease Aβ production. Palmitoylation targets *pal*APP to the detergent resistant lipid raft membranes (DRMs), and *pal*APP-dimers exhibit β-cleavage in the DRMs.

A complete loss of APP palmitoylation by 100μg/ml cerulenin resulted in pronounced loss of APP-APP dimerization, but not that of APP-CTFs (Fig 6B). This points to the fact that *pal*APP is predominantly dimerized. Accordingly, *pal*APP was found to form ~4.5 fold increased dimers compared to *tot*APP. In addition, *pal*APP exclusively formed *cis-*oriented dimers, which is a preferred orientation of APP-dimers for β-cleavage. We also found that *pal*APP dimers undergo β-cleavage in lipid-rich DRMs, which are one of the critical microdomains for amyloidogenesis. In conclusion, majority of *pal*APP appears to form *ci*s-dimers undergoing β-cleavage. Although we cannot entirely exclude the possibility of a pool of monomeric *pal*APP, our data overwhelmingly supports the conclusion that *pal*APP in its *cis*-dimerized form is a potential drug target for AD treatment. Identification of specific small molecule modulators for *pal*APP-dimers may become an effective targeted therapeutic strategy to lower Aβ in AD brains.

## Materials and Methods

### Cell culture and transfection

CHO_APP_, and naïve CHO cells were maintained and transfected with expression plasmids as described before [40, 41]. CHO cells stably expressing APP (CHO_APP_) were maintained in DMEM containing 10% serum supplemented with G418. Typically, 1.2 X 10^6^ cells were used for transfection and palmitoylation assays.

### Maintenance of immortalized hNPC cell line ReNcell VM (ReN cells)

ReN cells were maintained as described by Kim, Y.H. *et all* [32]. Briefly, ReN cells (Millipore) were maintained in Proliferation medium (484.5 ml DMEM/F12 (Gibco/Life Technologies) with 0.5 ml of heparin (2 mg/ml stock, STEMCELL Technologies), 10 ml of B27 (Life Technologies) 5 ml of 100X penicillin/streptomycin/amphotericin B (Lonza), 80 μl of bFGF stock and 100 μl of EGF stock) on Matrigel (Sigma-Aldrich) coated flasks at 37°C CO_2_ incubator. For differentiation the media were changed to Differentiation media, which is Proliferation media containing no growth factors, bFGF or EGF. The cells were maintained in Differentiation media for ~ 6 days to obtain neuronal structure prior to co-IP assays.

### Lentiviral infection of ReN cells

To transfect the ReN cells with the lentiviral constructs containing APP_mGFP_ and APP_mCherry_ expression plasmids (very generous gifts from Dr. Inna Slutsky, Sackler Faculty of Medicine, Tel Aviv University, Israel), we obtained the lentiviral vectors packaged by MGH viral core fascility. 1 X 10^6^ viral particle was used to infect 85% confluent proliferating ReN cells in 6-well dishes. After 24 h the cells were washed three times to stop the infection. The expression of the infected genes was confirmed by mGFP or mCherry GFP expression by fluorescence microscopy and western blot analysis. To probe APP_mGFP_ expression, anti-mGFP (Abcam, USA) antibody was used in immunostaining that specifically detects mGFP epitope. For APP_mCherry_ detection we used anti-mCherry antibody from Abcam.

### FACS enrichment of the transfected ReNcells

The infected ReNcells were washed with PBS and then incubated with Accutase (Millipore) for 5 min. The cell pellets were resuspended in PBS supplemented with 2% serum replacement solution (Life Technologies) and 2% B27, and then passed through a cell strainer filter (70 mm Nylon, BD Biosciences). The cell concentrations were adjusted to ~200,000 cells per ml and then enriched by using FACSAria cell sorter (MGH core facility, Charlestown, MA). GFP and/or mCherry channels were used to detect the expression of the transfected genes in the individual cells. The sorted/enriched cells were maintained in normal proliferation media. To sort CHO cells co-expressing APP_mGFP_+APP_mCherry_ similar procedure was used after 24h transfection of these cells with APP_mGFP_ and APP_mCherry_ expression plasmids using Effectene reagents following the manufacturer’s protocol.

### Expression plasmids and antibodies

C-terminally V5-epitope tagged APP_751_ (APP_wt-V5_) APP(C^186,187^S/A), APP(C^186^S), and APP(C^187^S) were used before [40]. Expression vectors encoding HA-epitope tagged DHHC-1, -7 and -21 (HA-DHHC-1, HA-DHHC-7 and HA-DHHC-21) were kind gifts from Dr. Masaki Fukata, NIPS, Okazaki [42]. Expression vector encoding HA-APP_Y_ was a kid gift from Dr. Stephen Pasternak, Robarts Research Institute, Ontario, Canada. The following APP antibodies were used: C66 (APP C-term), 22C11 (APP N-term; Chemicon), anti-sAPPβ (IBL International) and 6E10 (Signet). Polyclonal antibody against BACE was obtained from Affinity BioReagents (Golden, Colorado). Antibodies against epitope tags: anti-V5 (Invitrogen), anti-myc and anti-HA (Cell Signaling). Antibodies against flotillin (anti-Flotillin, lipid rafts marker), mGFP (anti-mGFP) and mCherry (anti-mCherry) were obtained from Abcam.

### Western-blot analysis

Cell lysates were prepared by directly extracting cells in a buffer containing 10 mM Tris–HCl at pH 6.8, 1 mM EDTA, 150 mM NaCl, 0.25% NP-40, 1% Triton X-100, and a protease inhibitor cocktail (Roche, Basel, Switzerland), followed by centrifugation at 16,000g. For mABE assay, the lysis buffer was complemented with 10 mM tris(2-carboxyethyl)phosphate (TCEP, from Sigma) and 10 mM N-ethylmaleimide (NEM, from Thermo Scientific). Proteins (20–100 μg) were either subjected to immunoprecipitation, ABE assay or simply resolved on 4–12% gradient Bis–Tris gels (Invitrogen, Carlsbad, CA), depending on the individual experiment, as described. The blots were visualized by enhanced chemiluminescence (ECL). The images were captured by using BioMax film (Kodak, Rochester, NY) and quantified using QuantityOne software (Biorad).

### Acyl Biotinylation Exchange assay

This assay is based on the substitution of biotin for palmitoyl modifications through a sequence of three chemical steps described before [4]: unmodified cystein thiols are blocked with N-ethyl maleimide (NEM); palmitoylation thioesters are cleaved by hydroxylamine (+NH_2_OH); and finally, the newly exposed cyateinyl thiols are marked with thiol-specific biotinylating reagent (HPDP-biotin in our experiments). Biotinylated proteins are then affinity-purified (AP) with streptavidin–agarose beads and probed for the protein of interest [43, 44]. Briefly, cells were lysed with lysis buffer (LB: 50 mM Tris-HCl, pH 7.5, 150 mM NaCl, 5 mM EDTA) containing 1% SDS, 2% Triton X-100, 0.5% NP-40, protease inhibitors, 10 mM tris(2-carboxyethyl)phosphine (TCEP) (Sigma) and 10 mM N-ethylmaleimide (NEM) (Thermo Scientific). Equal amounts of proteins were precipitated by chloroform-methanol before 1 M NH_2_OH-treatment (untreated samples served as controls), HPDP-biotin addition and affinity purification with StreptAvidin agarose. The precipitates were either probed with an appropriate antibody or Streptavidin-HRP to detect palmitoylated proteins. In some cases cells were treated with palmitoylation inhibitors, 2-bromopalmitate (2-BP) and cerulenin (from Sigma) prior to ABE assay.

### Modified ABE assay (mABE assay)

This assay is based on a modification of the ABE assay as described earlier [4, 13]. Briefly, cells were lysed in lysis buffer (150 mM NaCl, 5 mM EDTA, 50 mM Tris-HCl, pH 7.4, 1% Triton X-100, protease inhibitors, 10 mM TCEP and 10 mM NEM. Aliquots of lysates were incubated with appropriate antibodies to immunoprecipitate APP or sAPP. Immunoprecipitated proteins bound to agarose beads were treated with 1 M NH_2_OH (pH 7.4) followed by incubation with Biotin-HPDP at 4°C for 2 h to label the reactive cysteine(s). A sample prepared in absence of NH_2_OH served as negative control. The beads were washed and immunoblotted with Steptavidin-HRP (Cell Signaling) to detect palmitoylation.

### APP and palAPP dimerization assays (Co-IP assay)

Cells were co-transfected with expression plasmids encoding HA-APP_Y_ and APP_V5_ were lysed and subjected to pulled-down assay where HA-APP_Y_ was precipitated with anti-HA antibody followed by immunoblotting with anti-V5 antibody to detect co-immunoprecipitation of APP_V5_. Reverse pull-down was also performed where APP_V5_ was first pulled-down using anti-V5 antibody followed by probing with anti-HA antibody to detect co-IP of HA-APP_Y_. Co-IP assays were performed either in absence or presence of palmitoylation inhibitors 2-bromopalmitate or cerulenin to test APP-APP dimerization Co-immunoprecipitation of HA-APP_Y_ with APP_V5_ suggested APP-APP dimerization. Similar co-IP assay was performed on cells co-expressing APP_mGFP_ and APP_mCherry_. Antibody specific for mGFP (anti-GFP) was used to pull-down APP_mGFP_ prior to probing with anti-mCherry antibody to detect APP_mGFP_-APP_mCherry_ dimerization.

To detectermine *pal*APP-dimerization, co-IPd samples from cells co-expressing HA-APP_Y_ and APP_V5_ were subjected to mABE assay as described above. Streptavidin-HRP detected *pal*HA-APP_Y_ and *pal*APP_V5_ at molecular weights ~150 and ~100, respectively. Identification of both bands indicated *pal*APP-dimerization.

### Assays for trans-dimerization of APP and of *pal*APP

To test for APP trans-dimerization, co-IP assay was performed in mixed cell culture where CHO cells expressing HA-APP_Y_ were co-cultured with Neuro-2A cells expressing APP_V5_. Briefly, the co-cultured cells were grown to confluency prior to incubation without or with 1mM cell-impermeable crosslinker 3.3’-dithiobis[sulfosuccinimidyl propionate] (DTSSP, Sigma) at 4°C to crosslink proteins at the cell surface as described by Soba et al. [20]. After lysis, APP_V5_ was pulled-down using an anti-V5 antibody prior to probing the precipitate with an anti-HA antibody to detect co-IP with APP_V5_ indicating APP-dimerization. We next subjected the co-IPed APP forms to mABE assay to detect palHA-APP_Y_ and/or palAPP_V5_.

### FLIM Imaging and analysis

FLIM imaging was carried out as described by Fogel, H. [27]. For imaging in CHO cells, cells were transfected with expression plasmids encoding mEGFP-tagged APP alone or together with expression plasmid encoding mCherry-tagged APP (APP_mGFP_ or APP_mGFP_ + APP_mCherry_, respectively). For imaging in neuronal progenitor RenVM, the cells were incubated with lentiviral particles containing expressionng vectors for APP_mGFP_ or APP_mGFP_ + APP_mCherry_ (the lentiviral vectors were gifts from Dr. Inna Slutsky, Tel Aviv University, Tel Aviv, Israel). Cells were sorted by a cell sorter to obtain homogenous cultures of APP_mGFP_-expressing or APP_mGFP_ + APP_mCherry_-expressing cells. The cells were fixed and the FLIM analysis was performed as described previously [45]. Briefly, pulsing Chameleon Ti:Sapphire laser (Coherent Inc., Santa Clara, CA) was used to excite GFP donor fluorophore (two-photon excitation at 780 nm wavelength). The baseline lifetime (**t** 1) of the mGFP fluorophore (APP_mGFP_) was measured in the absence of the mCherry acceptor fluorophore (APP_mCherry_) (negative control, FRETabsent). Donor fluorophore lifetimes were recorded using a high-speed photomultiplier tube (MCP R3809; Hamamatsu, Bridgewater, NJ) and a fast time-correlated single-photon counting acquisition board (SPC-830; Becker & Hickl, Berlin, Germany). In the presence of the acceptor fluorophore, if the two fluorophores are <5–10 nm apart, FRET occurs and the donor fluorophore lifetime (t_2_) shortens. The acquired FLIM data were analysed using SPC image software (Becker & Hickel, Berlin, Germany) to fit the raw data from each pixel to multi-exponential fluorescence decay curves to calculate mGFP fluorescence lifetimes. The degree of donor life time *T*_*m*,_ was calculated (*T*_*m*_ *= (t1-t2)/t1*), where *t* is the fluorescence lifetime of the donor fluorophore (mGFP) measured in nanoseconds after pulse. To represent a “non-FRETing” population with a longer lifetime and a “FRETing” population with a shorter lifetime, the fluorescence lifetimes are plotted in a bar-graph as described by Fogel, H. et al [27].

### BiFC assay

Bio-immunofluorescence of split GFP constructs (BiFC) assay was performed as described before [17] with modification. Briefly, naïve CHO cells were transiently transfected (Lipofectamine 2000, Invitrogen) with plasmids encoding split-GFP APP, APP(1–10) and APP(11). The split-GFP plasmids were generous gifts from Dr. Claus U. Pietrzik from Department of Pathobiochemistry, University Medical Center of the Johannes Gutenberg-University Mainz, Germany. 24hs after transfection cells were sorted by a fluorescence activated cell sorter (FACS). Cells were sorted based on the fluorescence intensity to obtain a homogenous population of cells with equal BiFC signals. Approximately 100,000 cells were plated on coverslips on 12-well plates, and grown for 18 h. Cells were then treated with increasing amounts of cerulenin (0–100 μM) for 3 h before fixing in 4% paraformaldehyde in 1x PBS at room temperature (RT) for 30 min. Cells were washed with 1 x PBS for three times before mounting the coverslips on DAPI containing mounting media (ProLong Gold antifade with DAPI, Life Technologies). Fluorescence microscopy was performed under Nikon confocal microscope using 40X objective. The fluorescence intensity was measured by ImageJ software.

### Detergent Resistant Membrane (DRM) preparation

DRMs were purified as described in Navarro-Lerida *et al* [46] with modification. Briefly, ~2.5 X 10^5^ cells were resuspended in 5 volume (weight:volume) HEPES buffer (50 mM HEPES, pH 7.4, 0.15 M NaCl, 1 mM PMSF plus 0.5% Triton X-100) at 4°C. Cells were homogenized by passing through a syringe (0.5**x**16 mm) on ice for 10 times. The homogenate was brought up to 4 ml by adding 2 ml 80% sucrose in HEPES, and placed at the bottom of a Beckman SW40 Ultraclear tube. The discontinuous sucrose gradient (40-30-5%) was formed by sequentially loading 4 ml 30% sucrose and 4 ml 5% sucrose in HEPES. Cells fractions were separated by centrifugation at 200,000 g for 18 h in a SW40 rotor (Beckman) at 4°C. A light, scattered band confined to the 5–30% sucrose interface was observed that contained most flotillin, which is a subcellular marker for lipid raft-rich membranes. This fraction was collected as detergent resistant membranes or DRMs.

### In vitro BACE-activity assay

To test sAPPβ-sAPPβ dimers from APP-APP dimers upon BACE1 activity, we collected detergent resistant membrane (DRMs) from cells co-expressing HA-APP_Y_ and mycAPP. DRMs were mixed with 50mM Na-acetate buffer of pH 4 containing complete protease inhibitor mixture (Roche Applied Science), the aspartic protease inhibitor pepstatinA (10 M; Roche Applied Science), and the **γ** -secretase inhibitor **N** -[**N** -(3,5-difluorophenacetyl-L -alanyl)]-**S** phenylglycine **t** -butyl ester (10M; Calbiochem). BACE-activity was measured by incubating the mixture at 37°C. After 1 h incubation, the reaction was terminated by bringing the pH to 7.6. The samples were centrifuged at 100,000 g for 1 h to remove membranes, and the supernatant were subjected to immunoprecipitation with anti-myc antibody to IP myc-sAPPβ. The precipitate was probed with anti-HA antibody to detect co-IP of HA-sAPPβ suggesting sAPPβ-sAPPβ dimerization. DRMs were also collected from HA-APP_Y_+mycAPP expressing cells after treatment with increasing amounts of cerulenin or with 50 μM 2-bromopalmitate (2-BP). These DRMs were also subjected to in vitro BACE1-activity assay followed by co-IP experiment as described above.

### Pulse chase assay

CHO_APP_ cells were were metabolically labeled with 100 μM chemical palmitic acid probe, alkylene palmitic acid (Alkyl-C16; Invitrogen) as described previously [4]. After 6 h labeling cells were washed once with DMEM media, and incubated for 0.5–6 h at 37°C in DMEM supplemented with penicillin/streptomycin, 3.6 mg/ml fatty acid free BSA and 100 μM unlabeled palmitic acid (Sigma), as described before [47](Chen, C. and Manning, D. 2000). Cells were collected at 0.5, 1, 2, 3 and 6 h prior to IP with C66 antibody and subsequent labeling with TAMRA via Click-iT chemistry as we did before [4]. IPed samples were probed with anti-TAMRA antibody to detect *pal*APP.

### Half-life assay

CHO cells were transfected with BiFC APP (APP(1–10) expression plasmid. 24 h after transfection, cells were treated with 100 μM cyclohexamide and MG132 for 0–6 h as described before [48]. After treatment cells were lysed and equal amount of lysates were subjected to SDS-PAGE and immunobloted with C66, anti-mGFP, or anti-actin antibodies to detect the levels of endogenous APP, APP(1–10) and actin.

### Aβ_40_ and Aβ_42_ determinations

For Aβ determination, CHO_APP_ cells were grown in six-well plates (Becton Dickinson Labware) till 80–90% confluency. After washing the cells once with PBS the cells were layered with 1 ml media for 6 h before adding increasing amounts of cerulenin (0–100 μg/ml). After 6 h of cerulenin treatment the conditioned media were collected and immediately subjected to Aβ ELISA assay. The levels of secreted Aβ_**40**_ and Aβ_42_ in the condition media were quantified by standard sandwich ELISA using the commercially available Aβ ELISA kit (Wako Pure Chemical) as before [4]. Aβ levels (in pmol/L) were plotted against cerulenin concentrations.

### Statistical analysis

All statistical analyses used a two-tailed Student’s t-test or one-way ANOVA, followed by a *post hoc* Tukey’s test. Error bars represented in graphs denote the s.e.m. Significance was assessed at *p<0.05 and **p<0.01.

## Supporting Information

S1 Fig Cerulenin lowered Aβ level in dose-dependent manner. Aβ ELISA demonstrates reduction of both Aβ_40_ and Aβ_42_ levels in conditioned media from CHO_APP_ cells treated with 0–100 μg/ml cerulenin (cer) for 6 h.
(TIF)

S2 Fig Half-life of untagged APP, BiFC-tagged APP, and *pal*APP. A. Expression of APP and APP(1–10) reduced upon treatment with cyclohexamide (Cyclo) in a time-dependent manner exhibiting half-life of both untagged APP and BiFC tagged APP(1–10) as ~3 h. The lysates were also probed with anti-actin antibody. B. CHO_APP_ cells were metabolically labeled with chemically-labeled palmitic acid (Alkyl-C16) for 6 h followed by chasing with unlabeled free palmitic acid for 0.5–6 h, as indicated. After immuoprecipitation of APP with C66 antibody from the labeled cells, the precipitates were subjected to Click-iT assay to incorporate TAMRA on Alkyl-C16. Immunobloting the precipitates with anti-TAMRA antibody detected Alkyl-C16 labeled APP (*pal*APP) and showed half-life of *pal*APP to be ~3 h. The blot is the representation of duplicate experiments.
(TIF)

S3 Fig Fluorescence-activated cell sorting and differentiated ReN cells. A. ReN cells expressing APP_mGFP_+APP_mCherry_ via Lentiviral infection were subjected to FACS analysis at the MassGeneral Hospital core fascility (MGH. Charlestown). Only 8.1% cells expressed both APP_mGFP_+APP_mCherry_ (P3 polulation) compared to 14% expressing APP_mCherry_ (P4 population) and 12.8% expressing APP_mGFP_ (P5 population) alone. B. After sorting the P3 population from the infected cells (panel a), the cells were differentiated into neuronal cells (panel b) prior to co-IP analysis.
(TIF)
