# Correction: Palmitoylated APP Forms Dimers, Cleaved by BACE1

**DOI:** 10.1371/journal.pone.0299972

**Published:** 2024-02-29

**Authors:** Raja Bhattacharyya, Rebecca H. Fenn, Cory Barren, Rudolph E. Tanzi, Dora M. Kovacs

In Fig 1, there were two instances of Fig 1B, whereas the figure legend referred to the presence of a Fig 1C. In Fig 2, the authors provide the correct HA-APP_Y_ panel in Fig 2B. The published panel resulted from an inadvertent duplication of another gel, part of which correctly appears in lanes 2–4 of the HA-APP_Y_ panel in Fig 3A. In Fig 6, the authors provide the correct APP-mGFP and CTF-mGFP panels in Fig 6B. The published panel resulted from an inadvertent duplication of the middle panels in Fig 6A. An error in labeling the top and middle panels of Fig 6A has been corrected. The original underlying quantitative data for this article were not included with the published article, but are now presented with this notice in S2 File.

**Fig 1:**
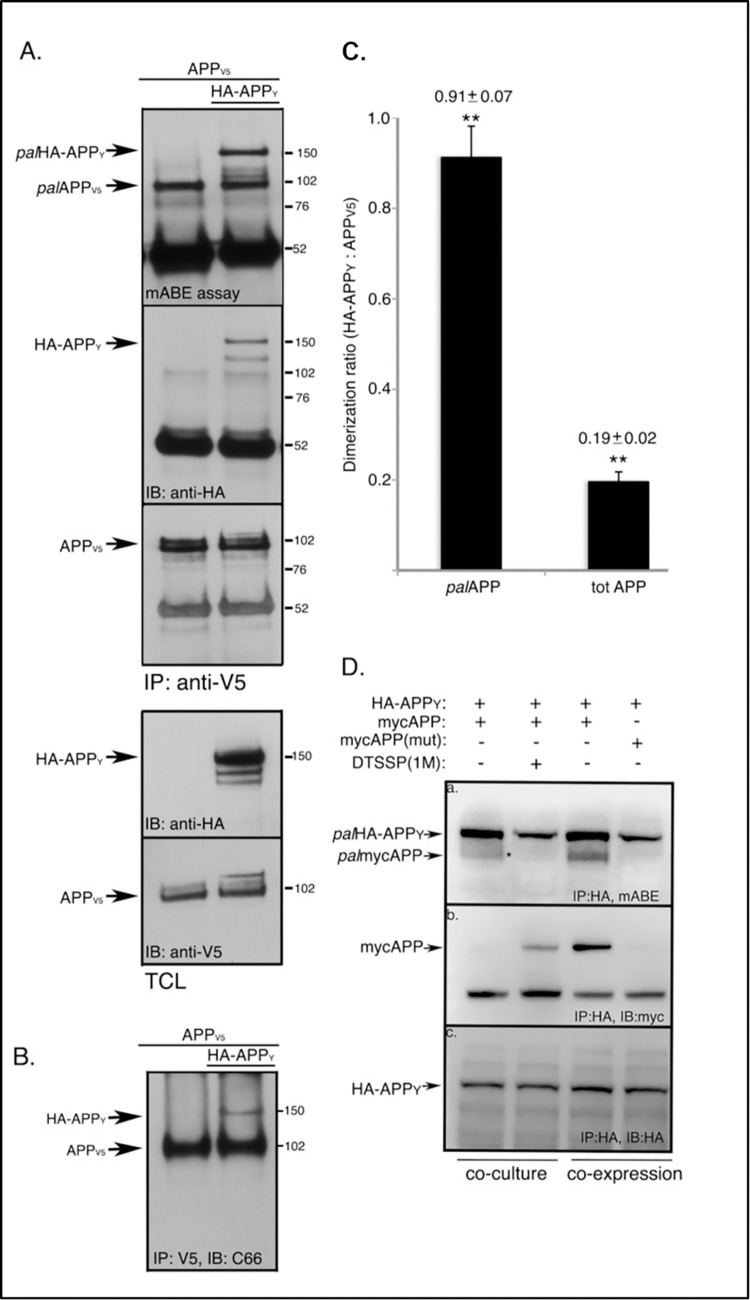
*pal*APP dimerizes ~4.5 times more efficiently compared totAPP and in cis-orientiation. A. Cells expressing APP_V5_ or APP_V5_ plus HA-APP_Y_ were subjected to co-immunoprecipitation assays to detect APP_V5_/HA-APP_Y_ interaction or APP-dimerization. APP_V5_ was immunoprecipitated with an anti-V5 antibody. Immunoprecipitates were probed with an anti-HA antibody to detect pull-down of HA-APP_Y_. Subsequently the immunoprecipitates were subjected to mABE assay to detect *pal*APP_V5_/HA-APP_Y_ interaction (or *pal*APP-dimerization). *Pal*APP_V5_ pulled down both *pal*APP_V5_ (M_wt_ ~102 kD) and *pal*HA-APP_Y_ (M_wt_ ~150 kD) from cells expressing APP_V5_ plus HA-APP_Y_ but not from cells expressing only APP_V5_. B. *Tot*APP-dimers (APP_V5_/HA-APP_Y_) only form in cells expressing both APP_V5_ and HA-APP_Y_. C. Quantitation of *pal*APP-dimers (*pal*APP_V5_/palHA-APP_Y_) versus *tot*APP-dimers (APP_V5_/HA-APP_Y_). Error bars show the s.e.m. (**p<0.01). D. *pal*APP dimerizes is *cis*-orientiation. Cells expressing HA-APP_Y_ and cells expressing mycAPP were co-cultured in absence or presence of 1mM cell-impermeable cross-linker DTSSP. Cell extracts were subjected to a pull-down assay, using an anti-HA antibody to immunoprecipitate HA-APP_Y_. To test for APP-dimerization, the precipitates were probed with an anti-myc antibody (*panel b*, *co-culture*). Cells co-expressing HA-APP_Y_ and mycAPP were also subjected to a co-IP assay using the anti-HA antibody to pull-down mycAPP with HA-APP_Y_.(*panel b*, *co-expression*). To detect *pal*APP-dimerization, the immunoprecipitates were also subjected to mABE assay to detect co-IP of *pal*HA-APP_Y_ with *pal*-mycAPP (*panel a*). The experiment is a representative of three independent experiments.

**Fig 2:**
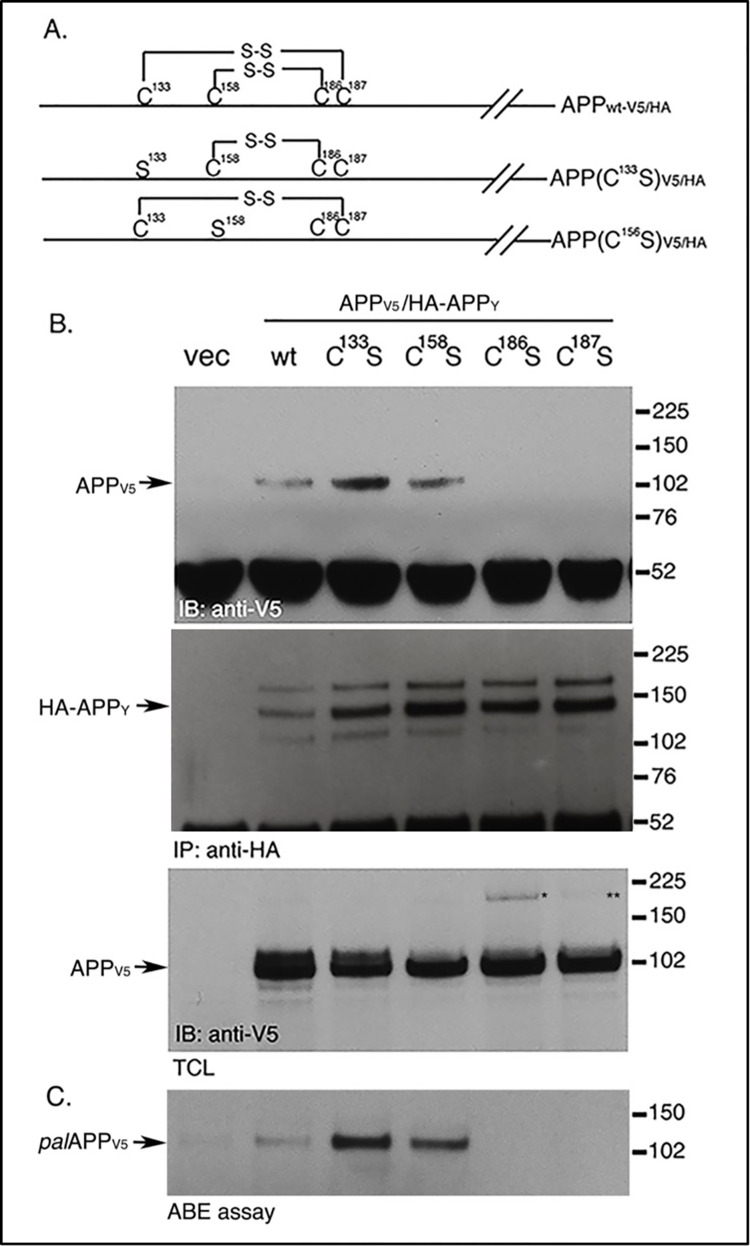
Palmitoylation-prone APP mutants exhibit increased APP dimerization compared to wtAPP. A. Schematic representation of the Cys to Ser mutants of APP used for the following co-immunoprecipitation assays. B. Co-immunoprecipitation assay in cells co-expressing APP_V5_ and HA-APP_Y_ and its mutants containing indicated Cys to Ser substitution. HA-APP_Y_ pulls down APP_V5_, indicating APP-APP dimerization. APP(C^133^S) and APP(C^158^S) show 2 fold increase in dimerization, while APP(C^186^S) and APP(C^187^S) fail to dimerize. APP(C^186^S) and APP(C^187^S) generated trace amounts of palmitoylation-independent dimers (* and **). C. ABE assay of cells overexpressing indicated APP mutants show 2 fold increased palmitoylation of APP(C^133^S) and APP(C^158^S), where as APP(C^186^S) and APP(C^187^S) were defective in palmitoylation.

**Fig 6:**
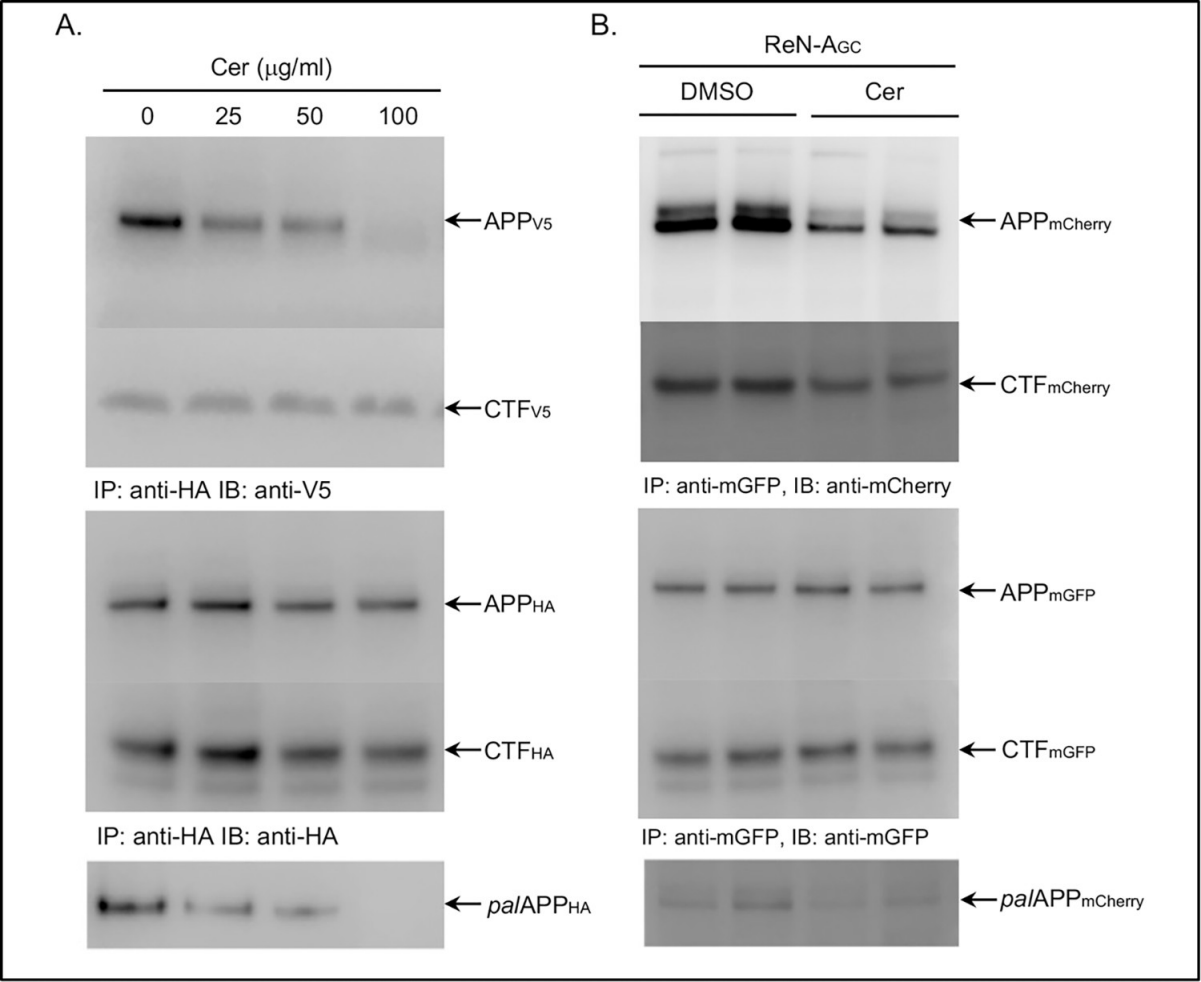
Palmitoylation inhibitors specifically impair ectodomain-dependent APP dimerization but not APP-CTF-dimerization. A. Naïve CHO cells co-expressing APP_V5_ and APP_HA_ were subjected to a co-IP assay in presence of DMSO (0 μg/ml) or increasing concentrations of cerulenin (25, 50 and 100 μg/ml). *fl*APPHA (APP_HA_) pulled down *fl*- as well as the C-terminal fragments of APP_V5_ (APP_V5_ and CTF_V5_, respectively) in DMSO-treated (0 μg/ml cerulenin) cells. In presence of cerulenin, co-IP of *fl*APP_V5_ (APP_V5_) with *fl*APP_HA_ (APP_HA_) decreased in a dose-dependent manner. Little or no co-IP of *fl*APP observed upon treatment with100 μg/ml cerulenin. In contrast, cerulenin had no effect on CTF_V5_ pull-down even at the highest concentration (100 μg/ml). Cerulenin reduced *pal*APP_HA_ levels in a dose-dependent manner (ABE assay) reaching complete inhibition at 100 μg/ml concentration. B. co-IP assay using an antibody specific for mGFP (anti-mGFP) to pull-down full-length (fl) APP_mGFP_ with APP_mCherry_ from differentiated neuronal cells (RenVM) co-expressing APP_mGFP_+APP_mCherry_. Anti-mGFP also pulled-down CTF_mCherry_ with CTF_mGFP_. Cerulenin (25 μg/ml) treatment of the cells prior to co-IP assay dramatically decreased *fl*APP_mGFP_-*fl*APP_mCherry_ interaction, but not that of CTF_mGFP_-CTF_mCherry_.

With this correction of this article [1], the authors provide updated versions of Fig 1, Fig 2, and Fig 6; original uncropped underlying blots for the figures of concern (S1 File); and the original underlying quantitative data for this article (S2 File).

The authors apologize for the errors in the published article.

## Supporting information

S1 File Raw Western blot data for Figs 2, 3, and 6. (PPTX)

S2 File Quantitative data for Figs 1, 3, 4, and 5. (PPTX)
