# Supplementary figures and images for: Palmitoylated APP Forms Dimers, Cleaved by BACE1
Source: PLoS One. 2016 Nov 22;11(11):e0166400. doi: 10.1371/journal.pone.0166400 (PMC5119739; doi:10.1371/journal.pone.0166400)

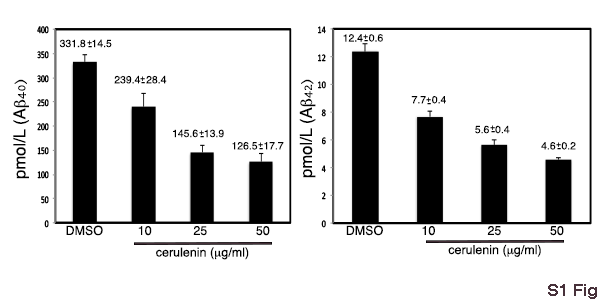

Supplement: S1 Fig — Aβ ELISA demonstrates reduction of both Aβ40 and Aβ42 levels in conditioned media from CHOAPP cells treated with 0–100 μg/ml cerulenin (cer) for 6 h. (TIF) [file pone.0166400.s001.tif]

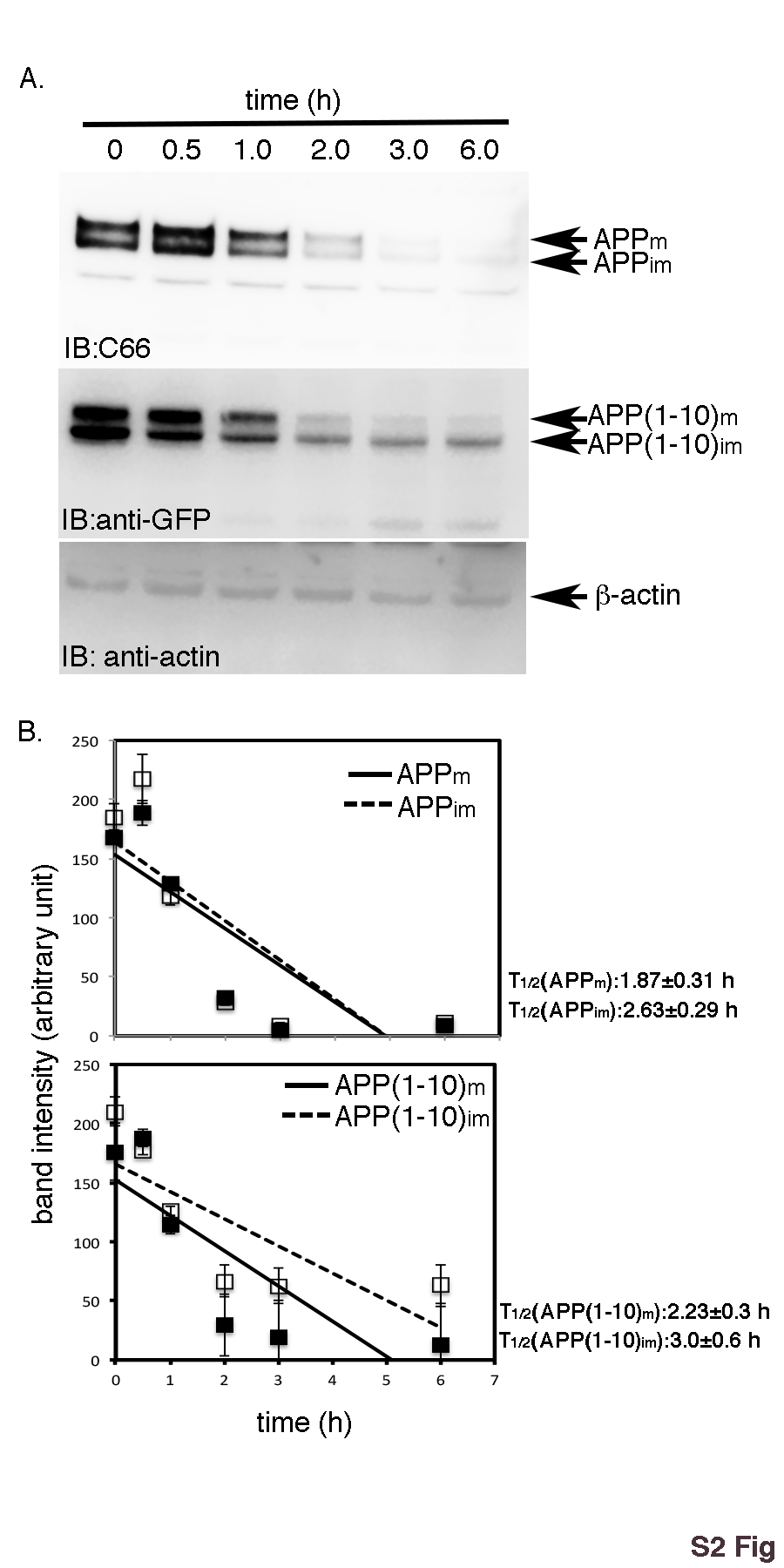

Supplement: S2 Fig — A. Expression of APP and APP(1–10) reduced upon treatment with cyclohexamide (Cyclo) in a time-dependent manner exhibiting half-life of both untagged APP and BiFC tagged APP(1–10) as ~3 h. The lysates were also probed with anti-actin antibody. B. CHOAPP cells were metabolically labeled with chemically-labeled palmitic acid (Alkyl-C16) for 6 h followed by chasing with unlabeled free palmitic acid for 0.5–6 h, as indicated. After immuoprecipitation of APP with C66 antibody from the labeled cells, the precipitates were subjected to Click-iT assay to incorporate TAMRA on Alkyl-C16. Immunobloting the precipitates with anti-TAMRA antibody detected Alkyl-C16 labeled APP (palAPP) and showed half-life of palAPP to be ~3 h. The blot is the representation of duplicate experiments. (TIF) [file pone.0166400.s002.tif]

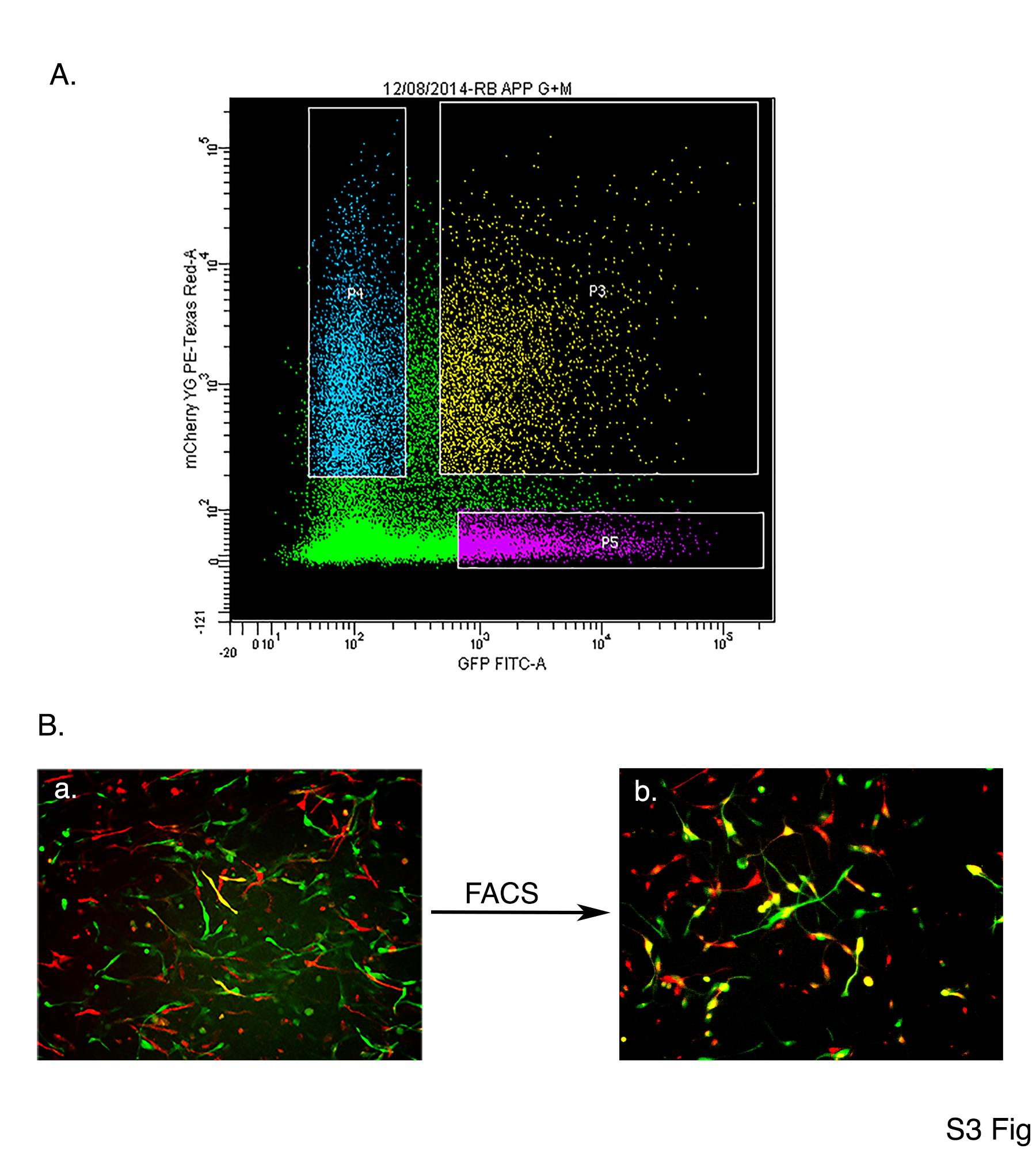

Supplement: S3 Fig — A. ReN cells expressing APPmGFP+APPmCherry via Lentiviral infection were subjected to FACS analysis at the MassGeneral Hospital core fascility (MGH. Charlestown). Only 8.1% cells expressed both APPmGFP+APPmCherry (P3 polulation) compared to 14% expressing APPmCherry (P4 population) and 12.8% expressing APPmGFP (P5 population) alone. B. After sorting the P3 population from the infected cells (panel a), the cells were differentiated into neuronal cells (panel b) prior to co-IP analysis. (TIF) [file pone.0166400.s003.tif]
