# Supplementary material for: Correction: Palmitoylated APP Forms Dimers, Cleaved by BACE1
Source: PLoS One. 2024 Feb 29;19(2):e0299972. doi: 10.1371/journal.pone.0299972 (PMC10903830; doi:10.1371/journal.pone.0299972)
Supplement: S1 File — (PPTX) [file pone.0299972.s001.pptx]

## Slide 1
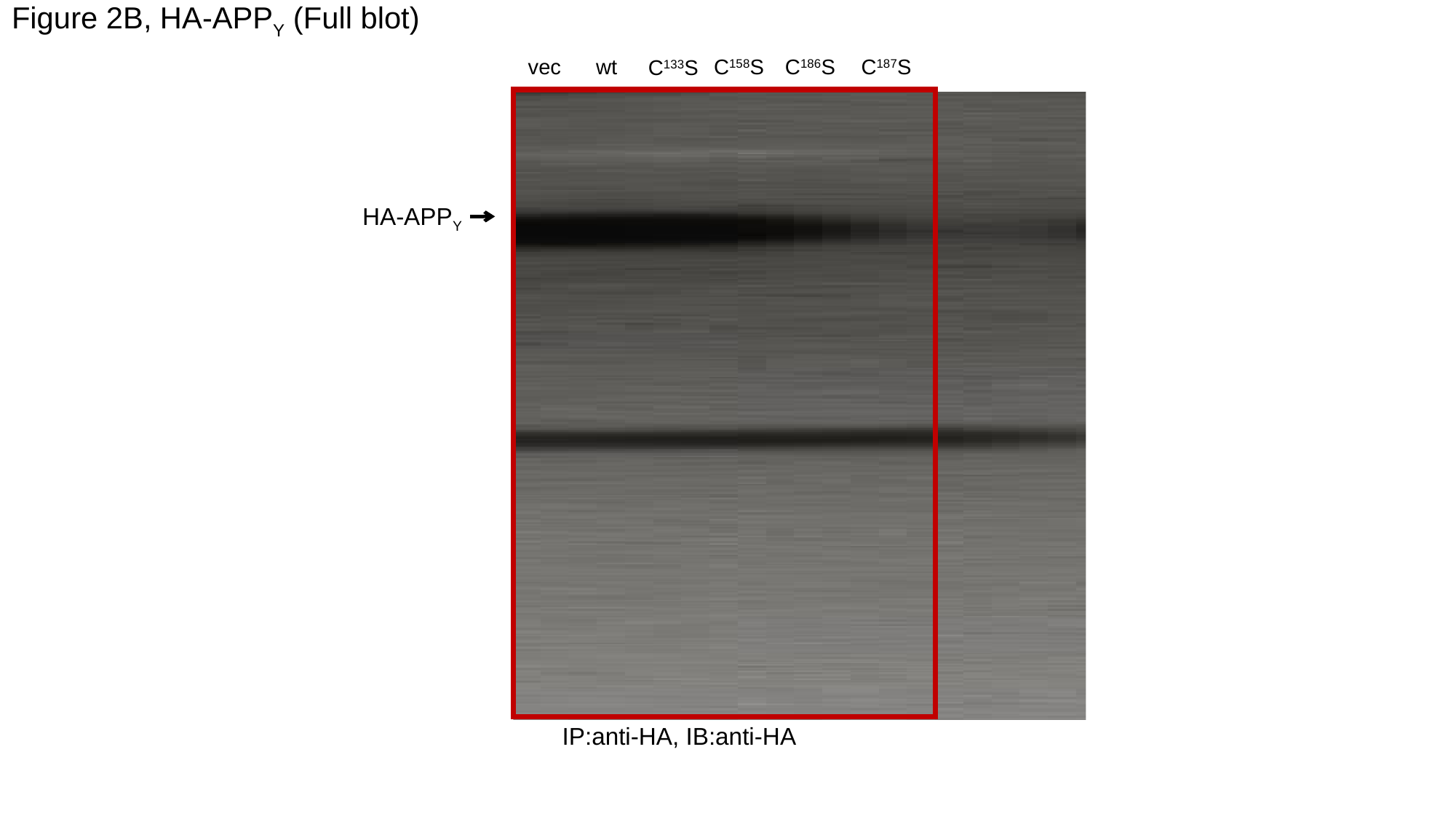

Figure 2B, HA-APPY (Full blot)
vec
wt
C158S
C186S
C187S
C133S
HA-APPY
IP:anti-HA, IB:anti-HA

## Slide 2
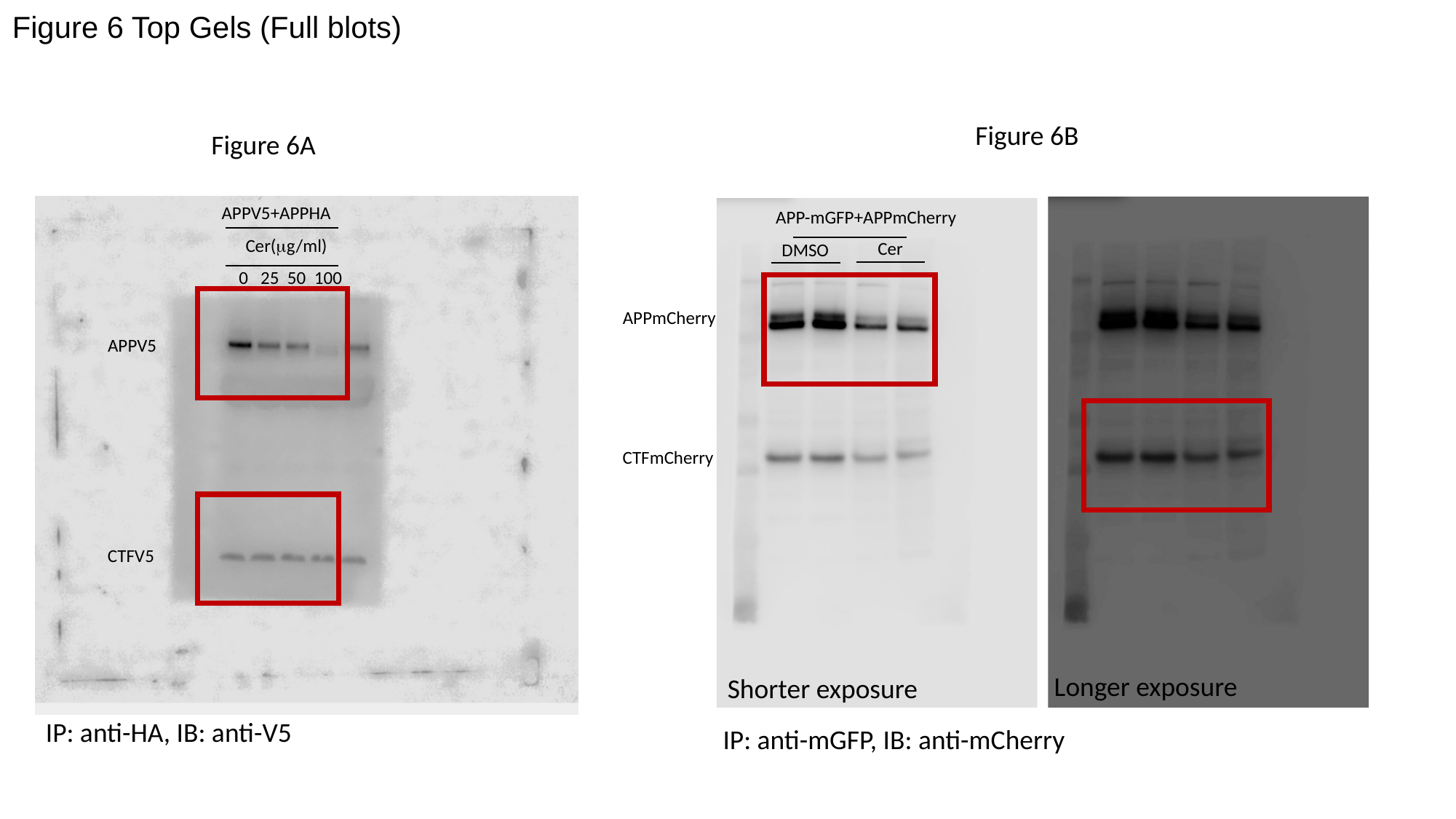

Figure 6 Top Gels (Full blots)
Figure 6B
Figure 6A
APPV5+APPHA
APP-mGFP+APPmCherry
Cer(mg/ml)
Cer
DMSO
0 25 50 100
APPmCherry
APPV5
CTFmCherry
CTFV5
Longer exposure
Shorter exposure
IP: anti-HA, IB: anti-V5
IP: anti-mGFP, IB: anti-mCherry

## Slide 3
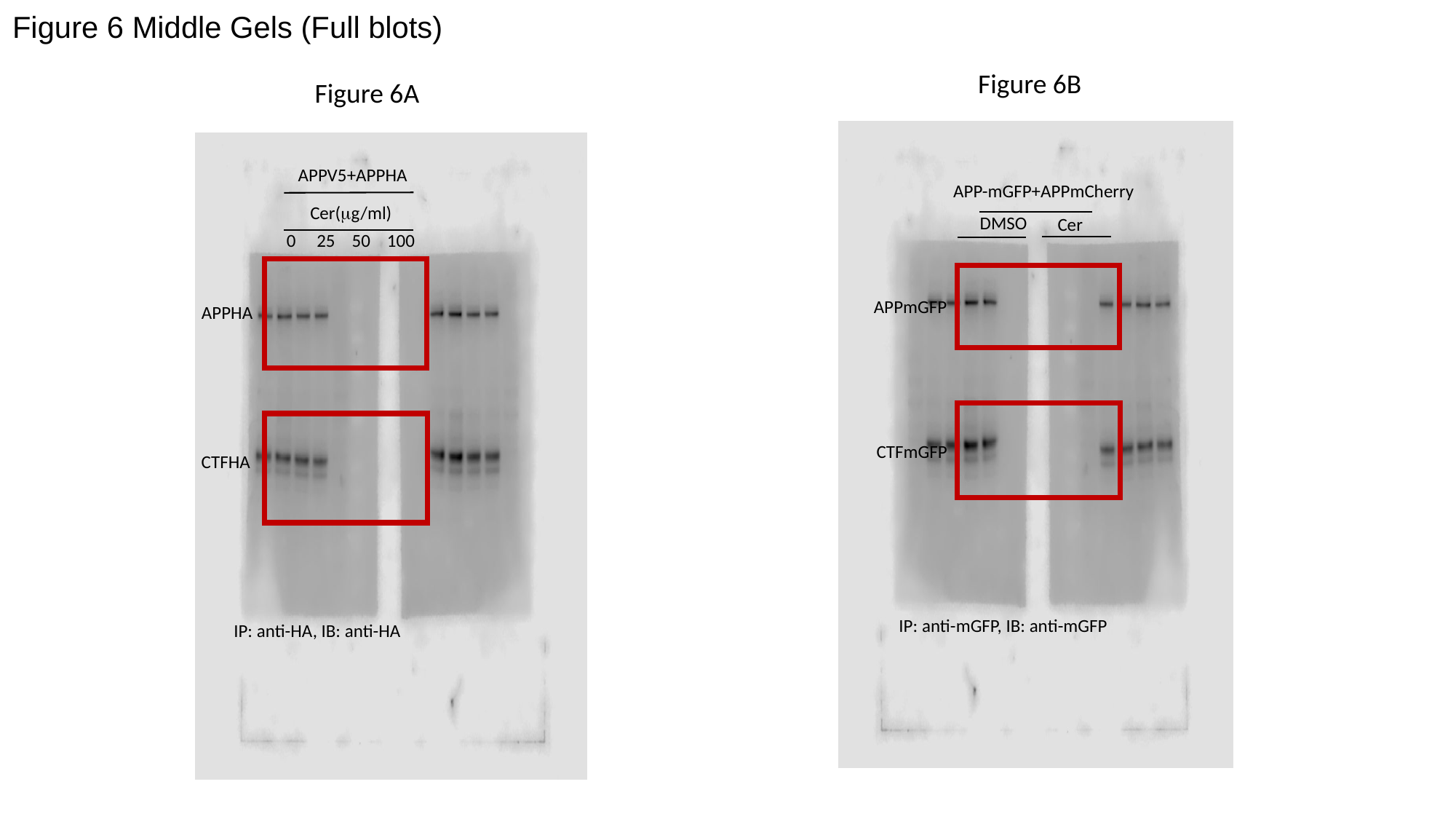

Figure 6 Middle Gels (Full blots)
Figure 6B
Figure 6A
APPV5+APPHA
APP-mGFP+APPmCherry
Cer(mg/ml)
DMSO
Cer
0 25 50 100
APPmGFP
APPHA
CTFmGFP
CTFHA
IP: anti-mGFP, IB: anti-mGFP
IP: anti-HA, IB: anti-HA
