# Supplementary material for: Correction: Palmitoylated APP Forms Dimers, Cleaved by BACE1
Source: PLoS One. 2024 Feb 29;19(2):e0299972. doi: 10.1371/journal.pone.0299972 (PMC10903830; doi:10.1371/journal.pone.0299972)
Supplement: S2 File — (PPTX) [file pone.0299972.s002.pptx]

## Slide 1
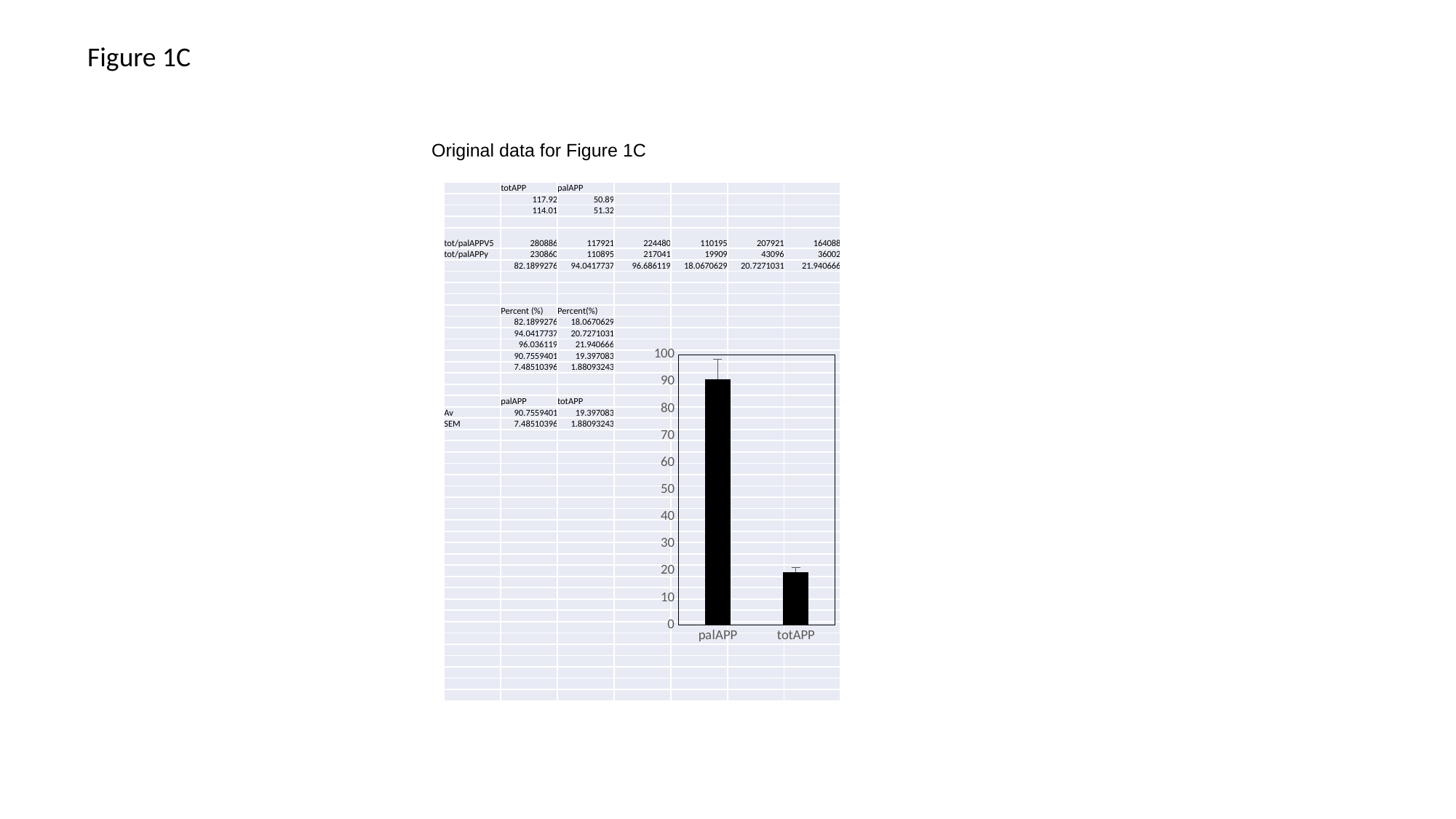

Figure 1C
Original data for Figure 1C
| | totAPP | palAPP | | | | |
| --- | --- | --- | --- | --- | --- | --- |
| | 117.92 | 50.89 | | | | |
| | 114.01 | 51.32 | | | | |
| | | | | | | |
| tot/palAPPV5 | 280886 | 117921 | 224480 | 110195 | 207921 | 164088 |
| tot/palAPPy | 230860 | 110895 | 217041 | 19909 | 43096 | 36002 |
| | 82.1899276 | 94.0417737 | 96.686119 | 18.0670629 | 20.7271031 | 21.940666 |
| | | | | | | |
| | | | | | | |
| | | | | | | |
| | Percent (%) | Percent(%) | | | | |
| | 82.1899276 | 18.0670629 | | | | |
| | 94.0417737 | 20.7271031 | | | | |
| | 96.036119 | 21.940666 | | | | |
| | 90.7559401 | 19.397083 | | | | |
| | 7.48510396 | 1.88093243 | | | | |
| | | | | | | |
| | | | | | | |
| | palAPP | totAPP | | | | |
| Av | 90.7559401 | 19.397083 | | | | |
| SEM | 7.48510396 | 1.88093243 | | | | |
| | | | | | | |
| | | | | | | |
| | | | | | | |
| | | | | | | |
| | | | | | | |
| | | | | | | |
| | | | | | | |
| | | | | | | |
| | | | | | | |
| | | | | | | |
| | | | | | | |
| | | | | | | |
| | | | | | | |
| | | | | | | |
| | | | | | | |
| | | | | | | |
| | | | | | | |
| | | | | | | |
| | | | | | | |
| | | | | | | |
| | | | | | | |
| | | | | | | |
| | | | | | | |
| | | | | | | |
### Chart
| Category | |
|---|---|
| palAPP | 90.75594011559973 |
| totAPP | 19.397083008155324 |

## Slide 2
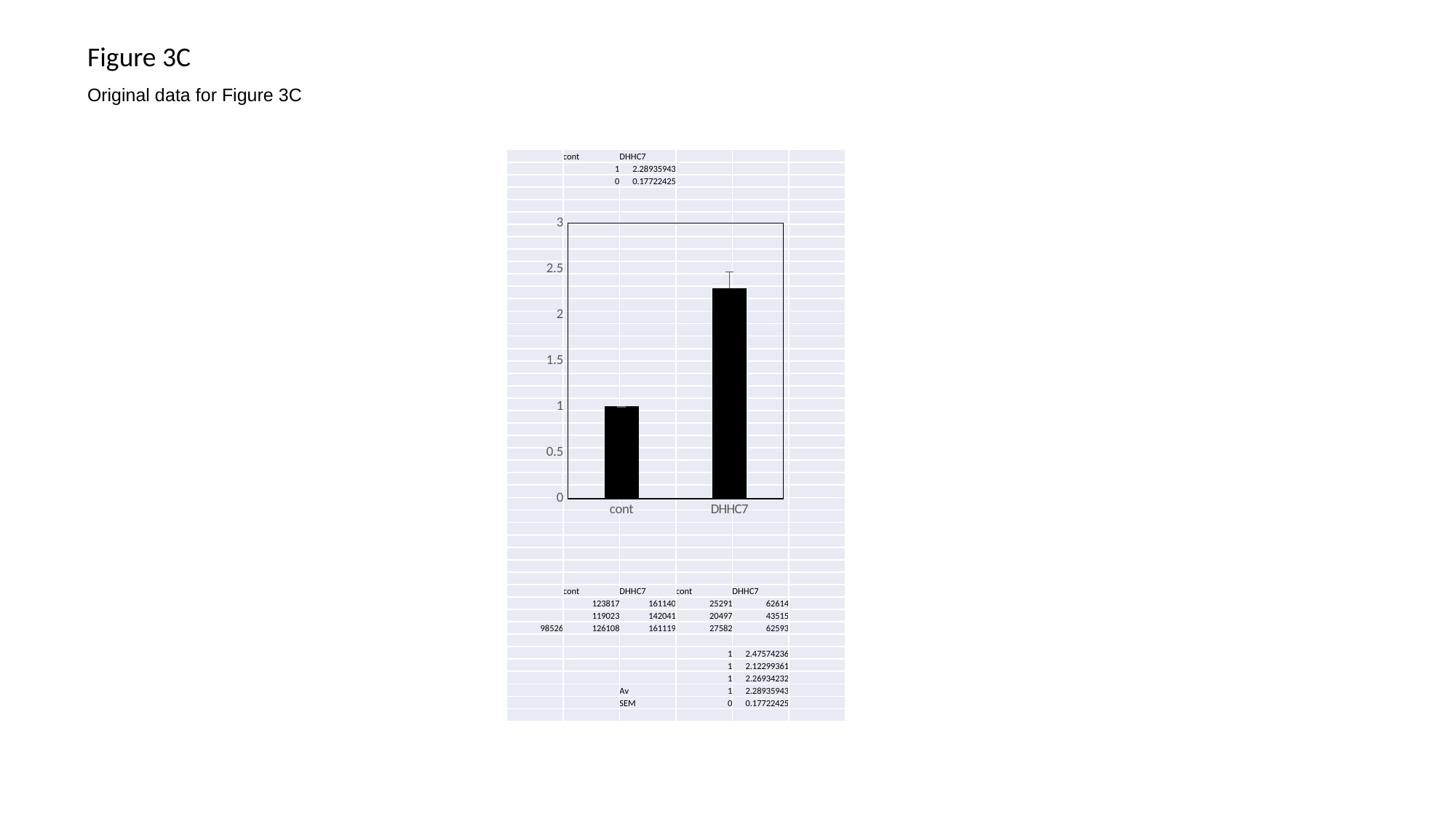

Figure 3C
Original data for Figure 3C
| | cont | DHHC7 | | | |
| --- | --- | --- | --- | --- | --- |
| | 1 | 2.28935943 | | | |
| | 0 | 0.17722425 | | | |
| | | | | | |
| | | | | | |
| | | | | | |
| | | | | | |
| | | | | | |
| | | | | | |
| | | | | | |
| | | | | | |
| | | | | | |
| | | | | | |
| | | | | | |
| | | | | | |
| | | | | | |
| | | | | | |
| | | | | | |
| | | | | | |
| | | | | | |
| | | | | | |
| | | | | | |
| | | | | | |
| | | | | | |
| | | | | | |
| | | | | | |
| | | | | | |
| | | | | | |
| | | | | | |
| | | | | | |
| | | | | | |
| | | | | | |
| | | | | | |
| | | | | | |
| | | | | | |
| | cont | DHHC7 | cont | DHHC7 | |
| | 123817 | 161140 | 25291 | 62614 | |
| | 119023 | 142041 | 20497 | 43515 | |
| 98526 | 126108 | 161119 | 27582 | 62593 | |
| | | | | | |
| | | | 1 | 2.47574236 | |
| | | | 1 | 2.12299361 | |
| | | | 1 | 2.26934232 | |
| | | Av | 1 | 2.28935943 | |
| | | SEM | 0 | 0.17722425 | |
| | | | | | |
### Chart
| Category | |
|---|---|
| cont | 1.0 |
| DHHC7 | 2.289359430822412 |

## Slide 3
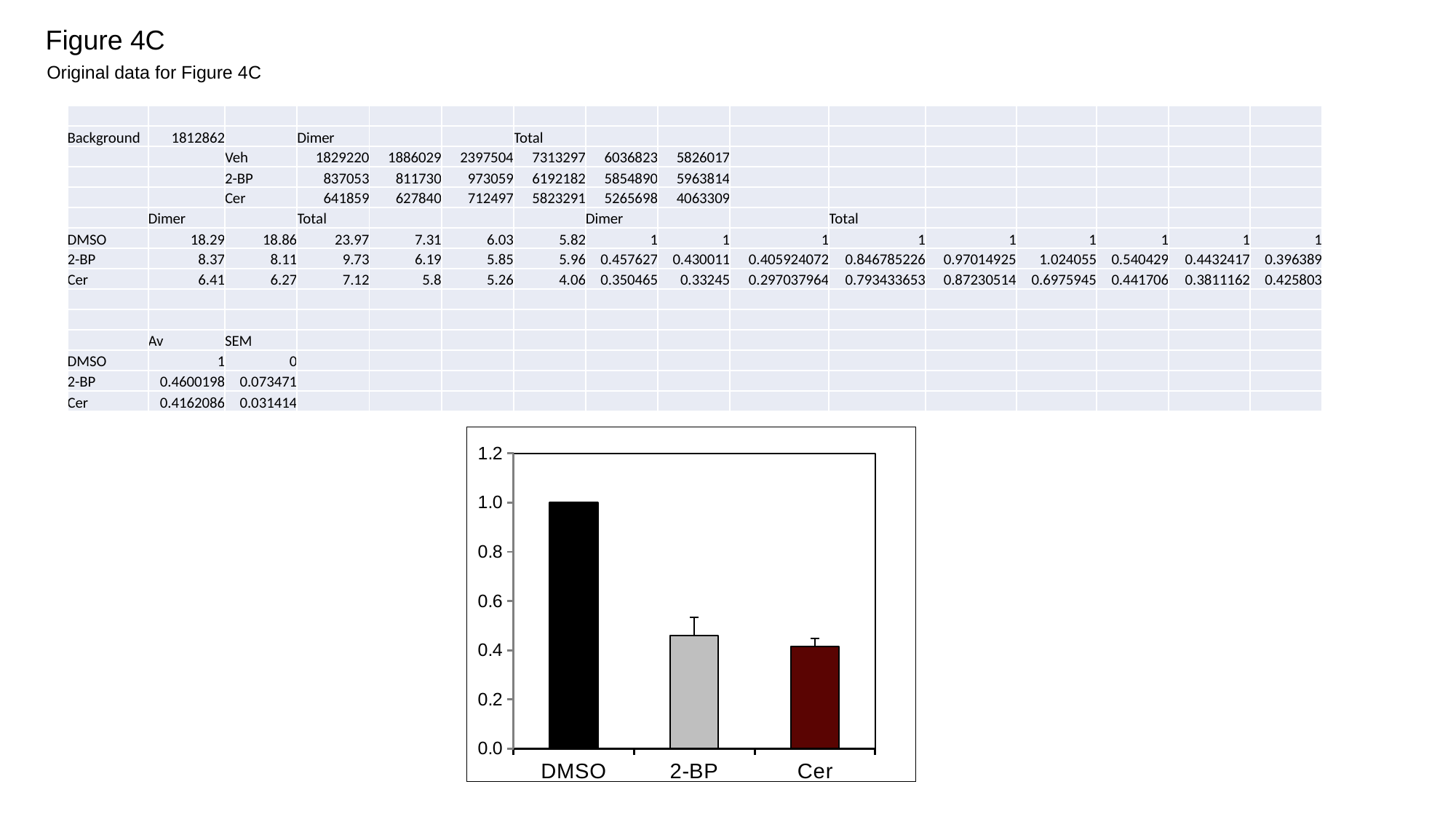

Figure 4C
Original data for Figure 4C
| | | | | | | | | | | | | | | | |
| --- | --- | --- | --- | --- | --- | --- | --- | --- | --- | --- | --- | --- | --- | --- | --- |
| Background | 1812862 | | Dimer | | | Total | | | | | | | | | |
| | | Veh | 1829220 | 1886029 | 2397504 | 7313297 | 6036823 | 5826017 | | | | | | | |
| | | 2-BP | 837053 | 811730 | 973059 | 6192182 | 5854890 | 5963814 | | | | | | | |
| | | Cer | 641859 | 627840 | 712497 | 5823291 | 5265698 | 4063309 | | | | | | | |
| | Dimer | | Total | | | | Dimer | | | Total | | | | | |
| DMSO | 18.29 | 18.86 | 23.97 | 7.31 | 6.03 | 5.82 | 1 | 1 | 1 | 1 | 1 | 1 | 1 | 1 | 1 |
| 2-BP | 8.37 | 8.11 | 9.73 | 6.19 | 5.85 | 5.96 | 0.457627 | 0.430011 | 0.405924072 | 0.846785226 | 0.97014925 | 1.024055 | 0.540429 | 0.4432417 | 0.396389 |
| Cer | 6.41 | 6.27 | 7.12 | 5.8 | 5.26 | 4.06 | 0.350465 | 0.33245 | 0.297037964 | 0.793433653 | 0.87230514 | 0.6975945 | 0.441706 | 0.3811162 | 0.425803 |
| | | | | | | | | | | | | | | | |
| | | | | | | | | | | | | | | | |
| | Av | SEM | | | | | | | | | | | | | |
| DMSO | 1 | 0 | | | | | | | | | | | | | |
| 2-BP | 0.4600198 | 0.073471 | | | | | | | | | | | | | |
| Cer | 0.4162086 | 0.031414 | | | | | | | | | | | | | |
### Chart
| Category | Av |
|---|---|
| DMSO | 1.0 |
| 2-BP | 0.4600198123072758 |
| Cer | 0.41620860507732704 |

## Slide 4
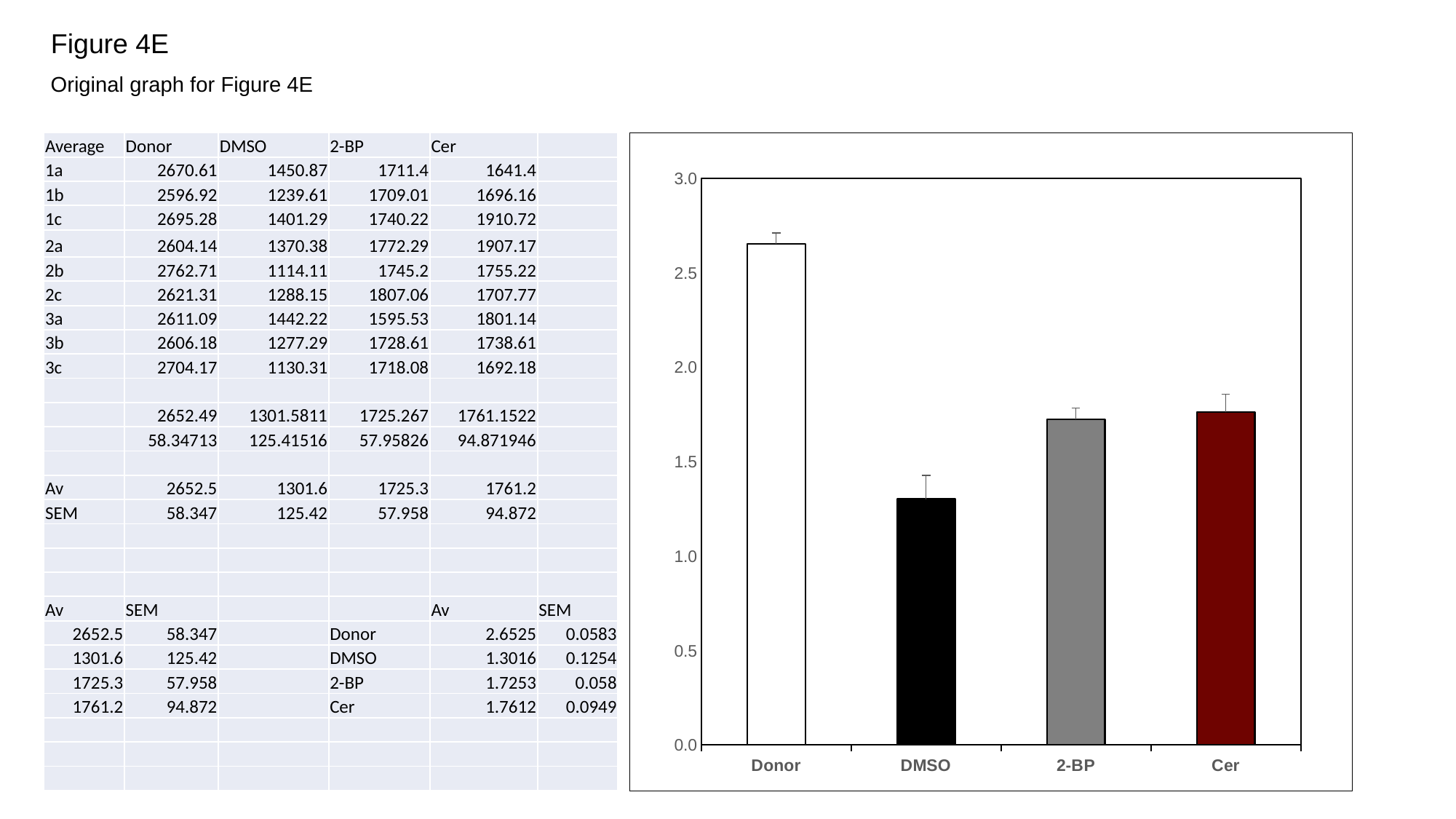

Figure 4E
Original graph for Figure 4E
| Average | Donor | DMSO | 2-BP | Cer | |
| --- | --- | --- | --- | --- | --- |
| 1a | 2670.61 | 1450.87 | 1711.4 | 1641.4 | |
| 1b | 2596.92 | 1239.61 | 1709.01 | 1696.16 | |
| 1c | 2695.28 | 1401.29 | 1740.22 | 1910.72 | |
| 2a | 2604.14 | 1370.38 | 1772.29 | 1907.17 | |
| 2b | 2762.71 | 1114.11 | 1745.2 | 1755.22 | |
| 2c | 2621.31 | 1288.15 | 1807.06 | 1707.77 | |
| 3a | 2611.09 | 1442.22 | 1595.53 | 1801.14 | |
| 3b | 2606.18 | 1277.29 | 1728.61 | 1738.61 | |
| 3c | 2704.17 | 1130.31 | 1718.08 | 1692.18 | |
| | | | | | |
| | 2652.49 | 1301.5811 | 1725.267 | 1761.1522 | |
| | 58.34713 | 125.41516 | 57.95826 | 94.871946 | |
| | | | | | |
| Av | 2652.5 | 1301.6 | 1725.3 | 1761.2 | |
| SEM | 58.347 | 125.42 | 57.958 | 94.872 | |
| | | | | | |
| | | | | | |
| | | | | | |
| Av | SEM | | | Av | SEM |
| 2652.5 | 58.347 | | Donor | 2.6525 | 0.0583 |
| 1301.6 | 125.42 | | DMSO | 1.3016 | 0.1254 |
| 1725.3 | 57.958 | | 2-BP | 1.7253 | 0.058 |
| 1761.2 | 94.872 | | Cer | 1.7612 | 0.0949 |
| | | | | | |
| | | | | | |
| | | | | | |
### Chart
| Category | |
|---|---|
| Donor | 2.65249 |
| DMSO | 1.30158111111111 |
| 2-BP | 1.72526666666667 |
| Cer | 1.76115222222222 |

## Slide 5
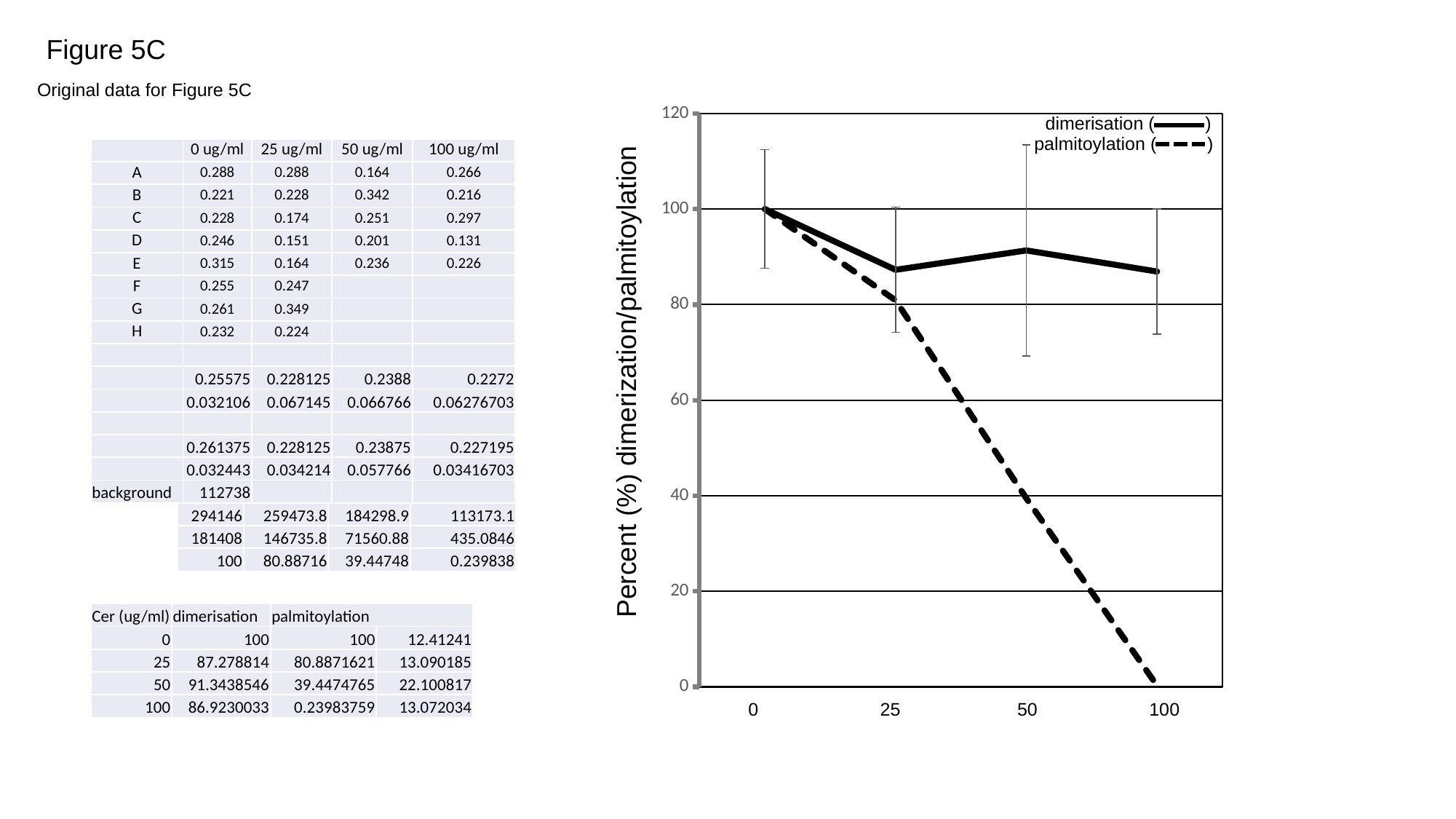

Figure 5C
Original data for Figure 5C
### Chart
| Category | | |
|---|---|---|dimerisation ( )
palmitoylation ( )
| | 0 ug/ml | 25 ug/ml | 50 ug/ml | 100 ug/ml |
| --- | --- | --- | --- | --- |
| A | 0.288 | 0.288 | 0.164 | 0.266 |
| B | 0.221 | 0.228 | 0.342 | 0.216 |
| C | 0.228 | 0.174 | 0.251 | 0.297 |
| D | 0.246 | 0.151 | 0.201 | 0.131 |
| E | 0.315 | 0.164 | 0.236 | 0.226 |
| F | 0.255 | 0.247 | | |
| G | 0.261 | 0.349 | | |
| H | 0.232 | 0.224 | | |
| | | | | |
| | 0.25575 | 0.228125 | 0.2388 | 0.2272 |
| | 0.032106 | 0.067145 | 0.066766 | 0.06276703 |
| | | | | |
| | 0.261375 | 0.228125 | 0.23875 | 0.227195 |
| | 0.032443 | 0.034214 | 0.057766 | 0.03416703 |
| background | 112738 | | | |
Percent (%) dimerization/palmitoylation
| 294146 | 259473.8 | 184298.9 | 113173.1 |
| --- | --- | --- | --- |
| 181408 | 146735.8 | 71560.88 | 435.0846 |
| 100 | 80.88716 | 39.44748 | 0.239838 |
| Cer (ug/ml) | dimerisation | palmitoylation | |
| --- | --- | --- | --- |
| 0 | 100 | 100 | 12.41241 |
| 25 | 87.278814 | 80.8871621 | 13.090185 |
| 50 | 91.3438546 | 39.4474765 | 22.100817 |
| 100 | 86.9230033 | 0.23983759 | 13.072034 |
0 25 50 100
